# Global Profiling of the Cellular Alternative RNA Splicing Landscape during Virus-Host Interactions

**DOI:** 10.1371/journal.pone.0161914

**Published:** 2016-09-06

**Authors:** Simon Boudreault, Camille Martenon-Brodeur, Marie Caron, Jean-Michel Garant, Marie-Pier Tremblay, Victoria E. S. Armero, Mathieu Durand, Elvy Lapointe, Philippe Thibault, Maude Tremblay-Létourneau, Jean-Pierre Perreault, Michelle S. Scott, Guy Lemay, Martin Bisaillon

**Affiliations:** 1 Département de biochimie, Faculté de médecine et des sciences de la santé, Université de Sherbrooke, Sherbrooke, Quebec, J1E 4K8, Canada; 2 Laboratoire de Génomique Fonctionnelle, Université de Sherbrooke, Sherbrooke, Quebec, J1E 4K8, Canada; 3 Département de microbiologie, infectiologie et immunologie, Faculté de médecine, Université de Montréal, Montreal, Quebec, H3C 3J7, Canada; Florida Atlantic University, UNITED STATES

## Abstract

Alternative splicing (AS) is a central mechanism of genetic regulation which modifies the sequence of RNA transcripts in higher eukaryotes. AS has been shown to increase both the variability and diversity of the cellular proteome by changing the composition of resulting proteins through differential choice of exons to be included in mature mRNAs. In the present study, alterations to the global RNA splicing landscape of cellular genes upon viral infection were investigated using mammalian reovirus as a model. Our study provides the first comprehensive portrait of global changes in the RNA splicing signatures that occur in eukaryotic cells following infection with a human virus. We identify 240 modified alternative splicing events upon infection which belong to transcripts frequently involved in the regulation of gene expression and RNA metabolism. Using mass spectrometry, we also confirm modifications to transcript-specific peptides resulting from AS in virus-infected cells. These findings provide additional insights into the complexity of virus-host interactions as these splice variants expand proteome diversity and function during viral infection.

## Introduction

Virus-host studies of a wide range of human viruses have identified many changes that occur in host cells upon viral infection, including modulation of host DNA/RNA/protein synthesis, induction of various anti-viral pathways, and sequestration/degradation of cellular proteins [1–3]. Viruses rely on host cell proteins and their associated mechanisms to replicate [4,5]. Numerous virus-host interactions therefore occur during infection, which enable both partners to respond to each other. Identifying the modifications that result from virus-host interactions is currently a crucial frontier in understanding viral infection.

Alternative RNA splicing is a central mode of genetic regulation found in higher eukaryotes which changes the sequence of an RNA transcript, thereby influencing gene expression on several levels [6]. Alternative splicing (AS) increases the variability of the cellular proteome by changing the composition of transcribed genes through differential choice of exons to be included in mature mRNAs. AS is therefore a critical determinant of protein diversity by producing multiple transcripts and, as a consequence, various proteins from a single gene [7,8]. Many splice variants have distinct and sometimes opposing functions. For instance, the Bcl-x RNA transcript can be alternatively spliced to produce two isoforms: Bcl-x(L), which has anti-apoptotic effects, and Bcl-x(S), which promotes apoptosis [9]. Not surprisingly, changes in pre-mRNA splicing patterns have been associated with many human diseases including cancer and amyotrophic lateral sclerosis [10–13]. The variety of alternative mRNA isoforms in the transcriptomes of higher eukaryotes suggests the presence of a complex interplay between *cis* elements and *trans* factors in order to regulate splicing decisions [14]. Splicing factors often bind specific pre-mRNA sequences to promote or repress splice-site recognition [15]. The role of some of these specific mRNA isoforms in disease biology is starting to emerge, and recent evidences indicate that some of these can be used as prognostic or diagnostic biomarkers [16–19]. The identification of molecules capable of correcting and/or inhibiting pathological splicing events is also an important issue for future therapeutic approaches [17,20,21].

Human viruses can utilize RNA splicing to facilitate the expression of their own genes. Modulation of AS of viral mRNA by viral-encoded factors is well established in such classical examples as papillomavirus, adenovirus and HIV, among others [22–24]. In contrast, the study of AS in mRNAs encoded by cellular genes during infection by human viruses remains sparse. Recent studies have shown that significant changes can be observed in the AS patterns of two cellular pre-mRNAs (i.e. PCBP2 and DST) as a result of the sequestration of the cellular HuR protein by Sindbis virus [25]. Similarly, the Poliovirus protease 2A (2Apro) induces a selective nucleo-cytoplasm translocation of several important RNA binding proteins and splicing factors which might lead to modifications in AS [26]. In the case of Epstein-Barr virus, a viral noncoding RNA (EBER1) interacts with splicing factor AUF1/hnRNP D, leading to a modification of cellular AS patterns [27]. In addition, previous studies reported the inhibition of pre-mRNA splicing by the Herpes simplex virus 1 (HSV-1) ICP27 protein. This viral protein is thought to contribute to HSV-1 host protein synthesis shut-off by interfering with cellular splicing machinery [28,29]. ICP27 interacts with components of the splicing machinery and causes a redistribution of splicing factors [30,31]. However, a recent transcriptomic study found no evidence of generalized inhibition of splicing upon HSV-1 infection [32].

In the present study, possible alterations to the global RNA splicing landscape of cellular genes upon viral infection were investigated using mammalian orthoreovirus, representative of a large family of viruses with a segmented double-stranded RNA genome, such as pathogenic rotaviruses. A non-pathogenic strain of mammalian itself is presently under clinical trials as a virotherapy agent against various cancers [33,34], while possible emergence of new pathogenic orthoreoviruses has been accumulating over the last few years [35]. There is thus major incentives to a better fundamental understanding of viral and cellular determinants that could affect reovirus replication and its effect on the host cells.

Our study reveals both the transcriptomic and AS landscapes of reovirus-infected cells. This study provides the first comprehensive portrait of global changes in the RNA splicing signatures that occur in eukaryotic cells following infection with a human virus. We identify modifications in 240 AS events of transcripts frequently involved in the regulation of gene expression and RNA metabolism. These findings provide additional insights into the complexity of virus-host interactions as these splice variants significantly expand proteome diversity and function during viral infection.

## Results

### Transcriptome of Reovirus-infected Cells

**Fig 1:**
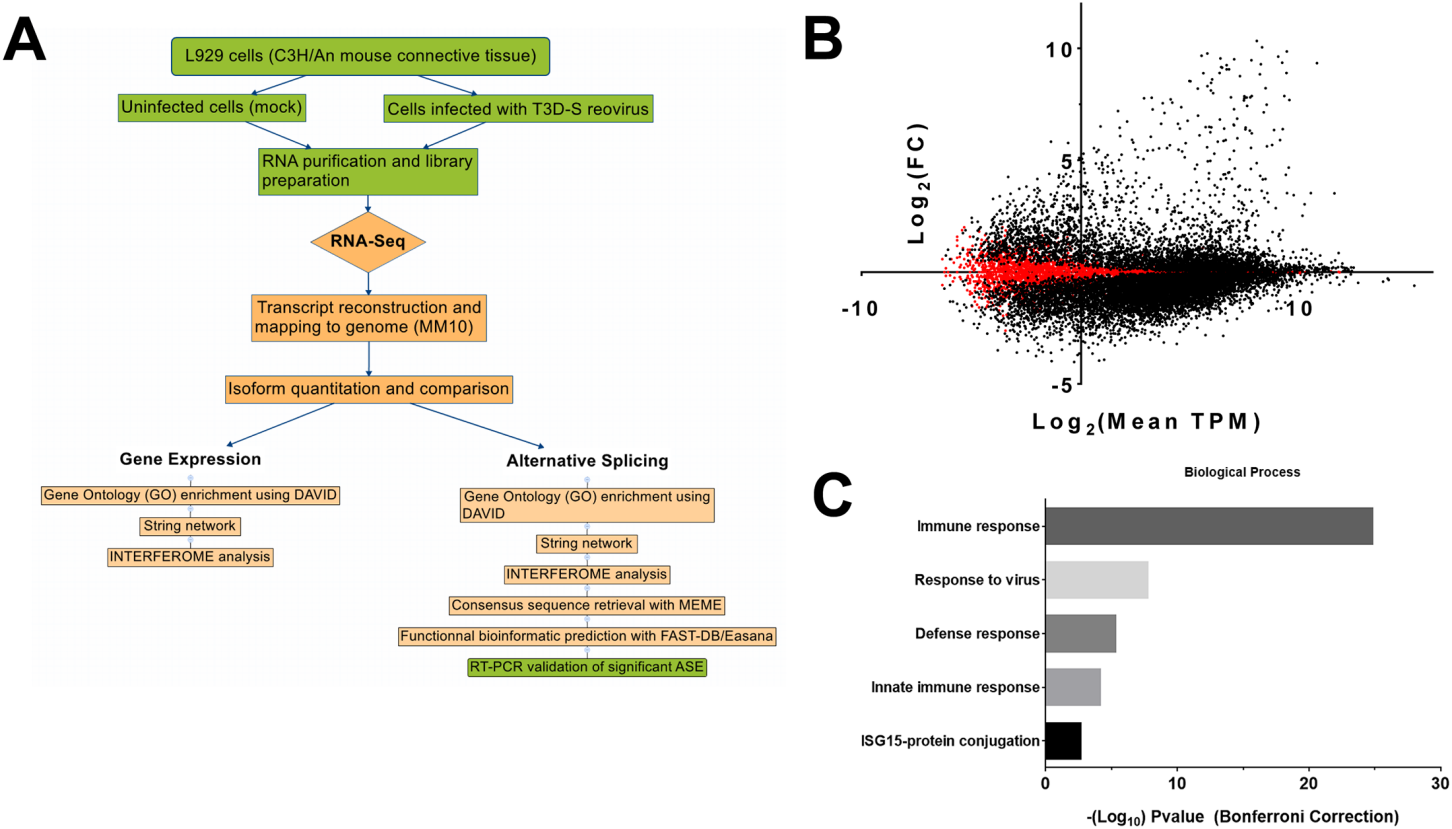
Transcriptomic studies of cells infected with reovirus. (A) Overview of the strategy used to identify the changes in both the cellular transcriptome and alternative splicing landscape upon reovirus infection. RNA-seq analysis was performed on infected cells at 14 hours post-infection. (B) MA-plot of cellular gene expression levels upon viral infection as compared to uninfected cells. The graph shows the fold-change (FC) in base 2 logarithm between infected and mock cells according to the mean expression of the gene in transcripts per million (TPM, also presented in log_2_). A cluster of over-expressed genes during viral infections with high TPM value can be seen on the upper right corner. (C) Gene ontology analyses of the 569 genes for which the expression was the most significantly modified following viral infection. Up- and down-regulated genes were imported into the DAVID gene ontology suite of programs at the NIAID. Ontological functions were determined for biological processes, and background of all detected genes was used.

Reoviruses have long served as a model system for studying viral pathogenesis and virus-host interactions [36]. In the present study, high-throughput RNA sequencing (RNA-seq) was used in order to analyze both the cellular isoform-level mRNA abundances and AS patterns that are altered during reovirus infection. Murine L929 fibroblasts were mock-infected or infected with mammalian orthoreovirus (serotype T3/Human/Ohio/Dearing/55). Although initially isolated from human, this virus strain is generally used as a representative of mammalian reoviruses that exhibit a very large host-range. The mouse is generally used as an animal model while the murine L929 cell line is most currently used for propagation and study of reovirus in cell culture; this cell line was thus chosen for studies presented herein. In order to capture AS changes preceding the cytopathic effect, RNA-seq was performed on infected cells at 14 hrs post-infection, and viral infection was confirmed by qRT-PCR using specific primers for three viral genes (Fig A in S1 Appendix). More than 132 million reads were obtained for each of the uninfected (mock-infected) and reovirus-infected cell samples (sequencing was done in triplicate, Table A in S1 Appendix). Reads were then mapped to the reference genome, followed by transcript assembly and analysis of cellular gene expression and RNA isoform abundance. The overview of all the analyses performed in this study is outlined in Fig 1A. The gene list (23,343 genes) was initially filtered to keep only data detected in at least two replicates for both virus-infected and mock-infected cells (16,044 genes). To ensure higher reproducibility, only genes with expression levels higher than one transcript-per-million (TPM) in either dataset were conserved. More than 12,500 genes were expressed at >1 TPM. Fold changes were then calculated between infected and uninfected cells (average TPM from the triplicate). Q-values (false-discovery rate) were calculated in order to correct for multiple statistical hypothesis testing, and results under 0.05 were considered significant. Our analysis revealed that the expression of a large number of cellular genes was modified upon viral infection (Fig 1B and Fig B in S1 Appendix). The 569 genes for which the expression was the most significantly modified (which represent 5% of genes with the highest fold-change) were used for further analysis. The complete list and the corresponding expression profiles of these 569 genes (380 up-regulated and 189 down-regulated) is presented in Fig C in S1 Appendix. As expected, gene ontology analysis revealed that the immune response is the most enriched function in the group of genes whose expression is upregulated upon infection (Fig 1C). Many of these immunomodulatory proteins (70/380, 18.4%) form a major interacting cluster and interact either directly or indirectly with each other (Fig D in S1 Appendix). It should be noted that a recent study by Schurch and Al showed that at least six replicates should be used in differential gene expression analysis [37]. However, they also concluded that genes with a fold change higher than two are well detected with three replicates. Since our aim was to focus on genes with the highest fold change, a number of three replicates is acceptable to validate the biological interpretation of our results.

### Modification of the Cellular AS Landscape Upon Viral Infection

**Fig 2:**
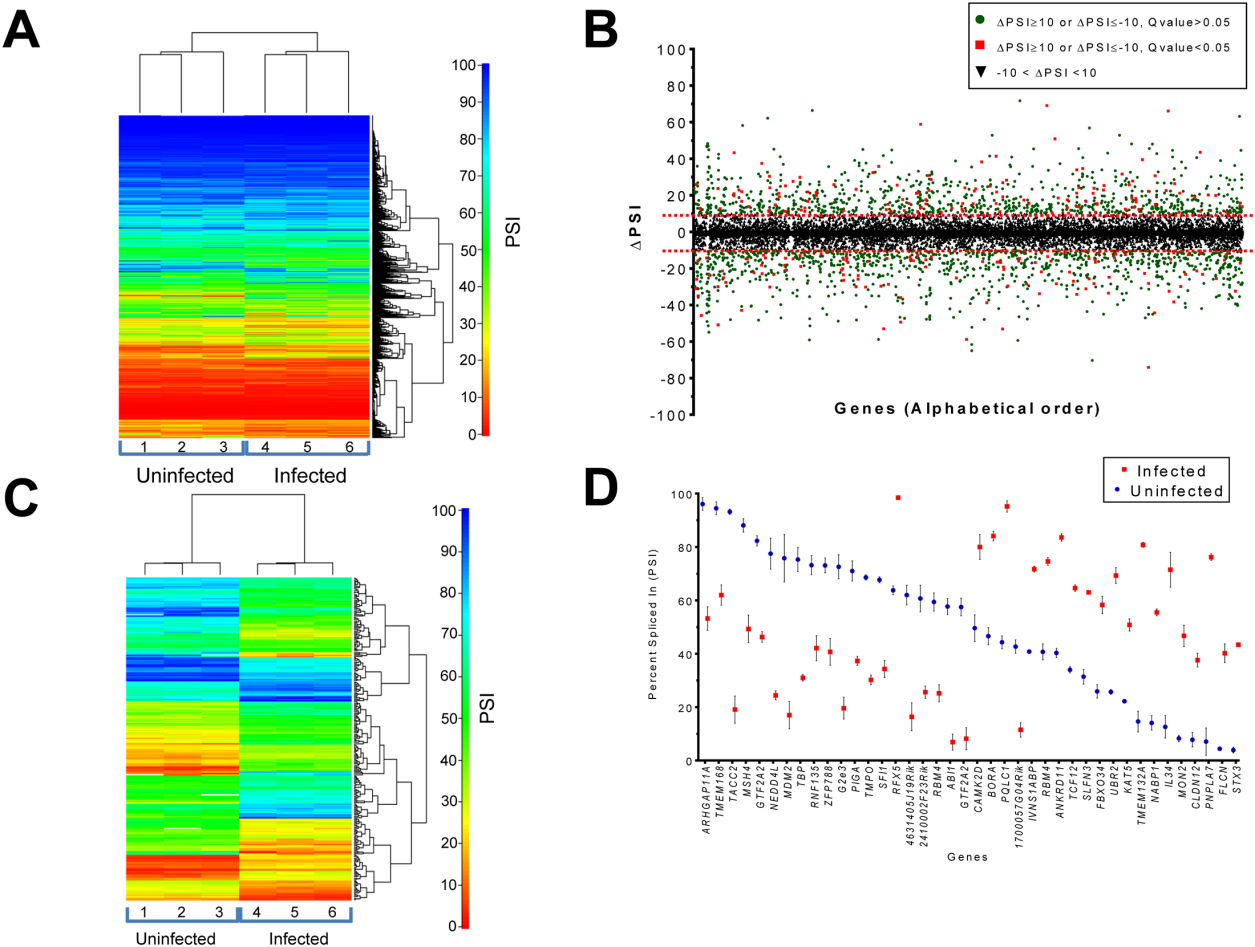
Global profiling of the cellular alternative splicing landscape and identification of differentially spliced ASEs during virus-host interactions. (A) Heatmap representation of isoform ratios for cellular transcripts in both infected and uninfected (mock) cells. RNA sequencing was done in triplicate for each condition. The map represents the percent-spliced-in (PSI) values based on isoform expression for the long and the short ASEs (see Materials and methods). Blue indicates high PSI values and red indicates low PSI values. (B) Alternative splicing events (ASEs) in cells infected with reovirus. ASEs were detected and quantified using the percent-spliced-in (PSI) metric. The graph shows an analysis of the difference in PSI values (Delta PSI) of the cellular genes following viral infection. Black triangles indicate Delta PSI values between -10 and 10, red squares indicate Delta PSI values greater than 10 or less than -10 with a Q value under 0.05, and green circles indicate same Delta PSI values but with a Q value above 0.05. (C) Heatmap representation of the 240 ASEs that are differentially spliced upon viral infection. RNA sequencing was done in triplicate for both the uninfected (mock) and infected cells. Blue indicates high absolute PSI values and red indicates low absolute PSI values. (D) PSI distribution of the 40 primary ASEs for which AS is the most significantly altered upon viral infection. The PSI values for the respective ASEs are indicated both for the uninfected and infected cells. Error bars indicate standard deviation.

**Fig 3:**
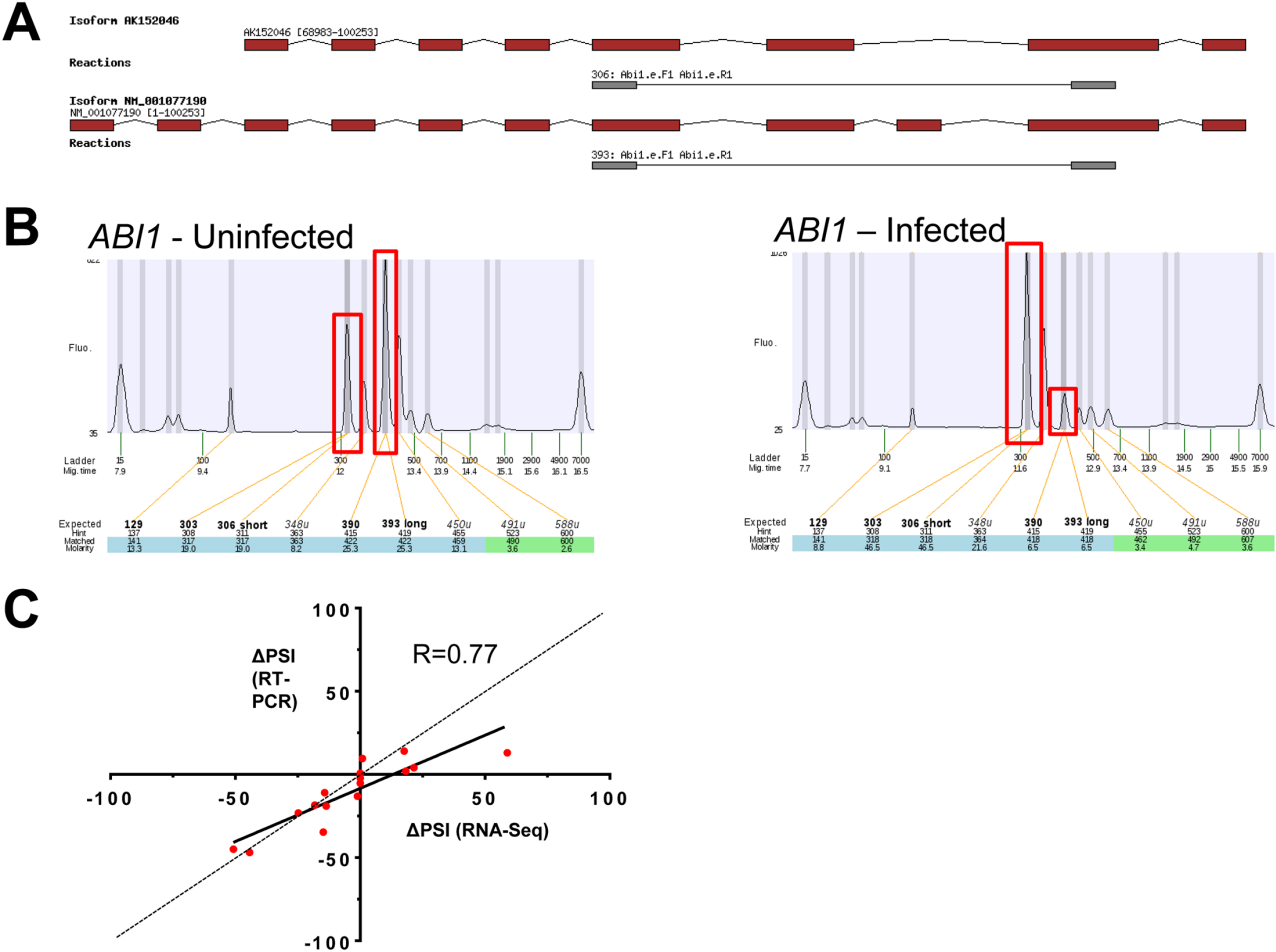
Validation of ASEs dysregulated in infected cells. (A) Overview of two isoforms encoded by the *ABI1* gene. Exons are depicted in red and the intervening introns are shown as thin black lines (not to scale). The primers used to detect the ASE by RT-PCR assays are shown in gray and the sizes of the expected amplicons (306 nt and 393 nt) are also indicated. The genomic coordinates of these two representative isoforms are also indicated. (B) Cellular mRNAs isolated from both uninfected and infected cells were analyzed by RT-PCR using specific primers to detect both forms of the modified ASE encoded by the *ABI1* gene. The amplified products were analyzed by automated chip-based microcapillary electrophoresis. Capillary electropherograms of the PCR reactions are shown. The positions and the amplitude of the detected amplicons are highlighted by red boxes. The positions of the internal markers are also indicated. The data shows the increase in the relative abundance of the short form (306 nt) and a decrease in the abundance of the long form (393 nt) upon viral infection. (C) Correlation between PSI values obtained from RNA-Seq and RT-PCR data. The analysis was performed on 16 selected ASEs (Abi1, Cwc22, Eif4a2, Hnrnpa2b1, Il34, Srsf3, Srsf5, Alkbh1, Cdkn2aip, Cflar, Hif1a, Mdm2, Serbp1, Sfswap, Smc2, Tbp). In all cases, the changes in AS levels detected by RT-PCR and the ones revealed through transcriptome sequencing displayed high levels of correlation (r >0.77). Sanger sequencing was also realized on several ASEs to confirm that RT-PCR reaction is specific and amplifies predicted ASE.

In order to identify the cellular AS patterns that are altered during reovirus infection, we evaluated modification to discrete splicing region on isoforms by quantifying all alternative splicing events (ASEs) using the percent spliced-in (PSI) metric. For every ASEs detected, quantitation was carried on based on the percentage on the long form on total form present for a specific ASE. (Fig 2A and 2B) (see Materials and methods). The ASE list (19,125 events) was initially filtered to keep only data detected in at least two replicates for both virus-infected and mock cells (8,482 genes). The ASEs that differed between mock-infected and infected cells with a P-value of less than 0.05 were conserved (1,732 events). To ensure higher stringency, the ASEs were further filtered with a cutoff Q-value of less than 0.05 (416 events). From these events, only those with a difference higher than 10% in |PSI| were considered biologically relevant. Using such an approach, we identified 240 splicing events belonging to 194 genes for which the AS pattern was significantly modified upon viral infection (Q <0.05, Delta|PSI| >10) (Fig 2C and Fig E in S1 Appendix) including 42 events which had Delta |PSI| values higher than 30% (Table B in S1 Appendix). To identify cellular pathways for the differential ASEs associated with viral infection, we performed a functional enrichment analysis on the 240 selected ASEs. The analysis revealed significantly enriched (P < 0.05, Bonferroni correction) terms in both RNA metabolism (22.3%) and gene expression (25.1%) (Fig F in S1 Appendix). A significant number of the modified ASEs are encoded by genes with important roles in viral infection/immunity. For instance, alterations in the splicing patterns of *RFX5*, which encodes for a protein involved in MHC-II expression, *MDM2*, a key cellular component of the signaling pathway used by reovirus for infection, and *RFN135*, which encodes a RING finger protein involved in the RIG-I/MDA5-mediated induction of IFN-alpha/beta pathways were observed upon viral infection. Through manual curation of functional annotations, we also identified 23 transcription factors, 7 proteases, 5 kinases, 23 hydrolases, and 9 splicing factors for which AS is significantly modified upon viral infection (Table 1). An example of a differentially spliced transcript is presented in Fig 3A and 3B, illustrating the modifications in isoform usage in transcripts encoded by the *ABI1* gene upon viral infection. Additional examples of alterations in splicing profiles are displayed in Fig G in S1 Appendix. The variations in AS of the 40 ASEs for which AS is the most significantly altered upon viral infection are presented in Fig 2D, and the complete list of the differential ASEs associated with viral infection is shown in Fig H in S1 Appendix. Finally, we used RT-PCR analysis in order to experimentally validate the differential ASEs that were observed through RNA-seq studies. Specific primers were designed to allow detection of sixteen predicted ASEs by PCR. Our results demonstrated that the changes in AS levels detected by RT-PCR were similar to the ones revealed through transcriptome sequencing, and displayed high levels of correlation (r = 0.77) (Fig 3C). Sanger sequencing was also realized on several ASEs to confirm that RT-PCR reactions are specific and amplify the predicted ASEs (Table C in S1 Appendix).

**Table 1:** Protein families found in the 240 transcripts that are differentially spliced upon viral infection.

Protein Classification	Frequency	Percentage
Calcium-binding protein (PC00060)	1	0.5%
Cell adhesion molecule (PC00069)	2	1,0%
Cell junction protein (PC00070)	1	0.5%
Chaperone (PC00072)	1	0.5%
Cytoskeletal protein (PC00085)	10	5.1%
Defense/immunity protein (PC00090)	3	1.5%
Enzyme modulator (PC00095)	23	11.7%
Extracellular matrix protein (PC00102)	2	1.0%
Hydrolase (PC00121)	19	9.7%
Kinase (PC00137)	5	2.6%
Ligase (PC00142)	10	5.1%
Membrane traffic protein (PC00150)	4	2.0%
Nucleic acid binding (PC00171)	47	24,0%
Oxidoreductase (PC00176)	2	1.0%
Phosphatase (PC00181)	2	1.0%
Protease (PC00190)	7	3.6%
Receptor (PC00197)	4	2.0%
Signaling molecule (PC00207)	2	1.0%
Splicing Factors	9	4.6%
Structural protein (PC00211)	1	0.5%
Transcription factor (PC00218)	23	11.7%
Transfer/carrier protein (PC00219)	3	1.5%
Transferase (PC00220)	11	5.6%
Transporter (PC00227)	4	2.0%

It should also be noted that we also identified the cellular AS pattern that is altered during infection with a mutant reovirus harboring a single amino acid substitution in the mRNA capping enzyme λ2 (P4L-12 mutant). This mutant has been previously shown to display an increase in interferon sensitivity [38,39]. Analysis of the ASEs revealed almost identical modifications to the cellular AS patterns than with the wild-type reovirus (Fig I in S1 Appendix). Indeed, upon infection with the mutant virus, we identified similar changes to the AS of cellular transcripts i.e. the AS of the same events were modified and displayed similar ΔPSI values. The only notable exception was for an ASE on the ATAT1 gene (Alpha Tubulin Acetyltransferase 1) which showed a considerable ΔPSI shift from WT virus (ΔPSI = 13.8) to mutant (ΔPSI = -10.4). However, RT-PCR validation failed to validate this shift, hence pointing toward a sequencing artifact rather than a mutant virus-specific change of alternative splicing.

### Characterization of the ASEs that are Modified Upon Viral Infection

**Fig 4:**
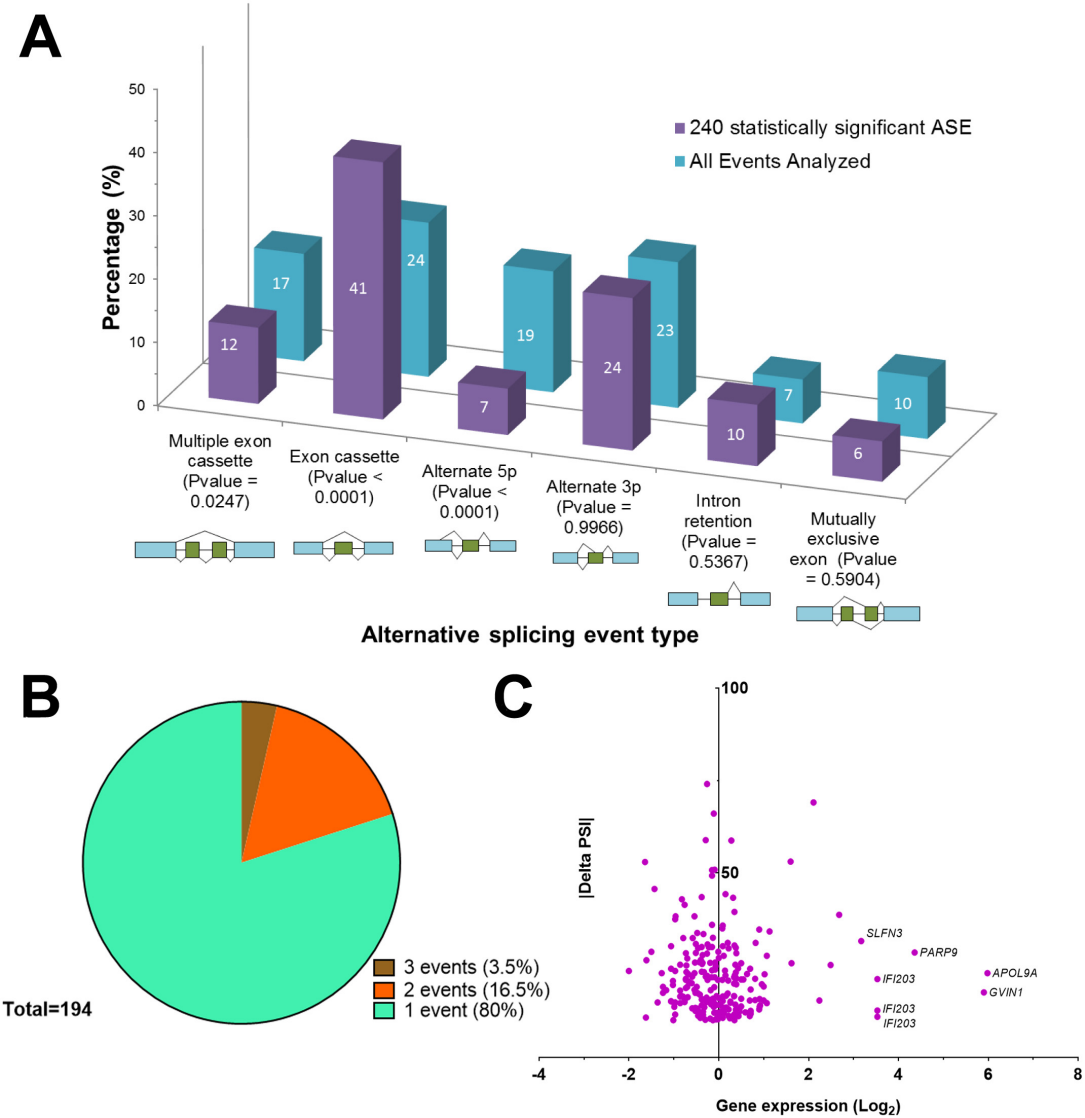
Characterization of the ASEs that are modified upon viral infection. (A) The percentage of splicing profiles found in cells and in the 240 differentially spliced transcripts are presented. Schematic representations of the various splicing profiles are also indicated as well as the statistical significance (chi-square with Yates' correction). (B) Distribution of the number of ASEs per gene. The vast majority of the ASEs that are modified upon viral infection were found at a level of one splicing event per gene. (C) Gene expression levels of the 240 differentially spliced transcripts. The graph displays both the modifications in gene expression and alternative splicing of the 240 ASEs that are differentially spliced upon viral infection. The variations in gene expression are presented in a logarithmic scale (Log_2_).

We next compared the profiles of the 240 selected ASEs that are differentially spliced upon viral infection against all the ASEs that were detected in our RNA-seq experiments. Among the different types of AS changes noted in the 240 selected ASEs, exon cassette events were the most common and represented a significant proportion of total changes (p < 0.0001; Fig 4A) as compared to all the ASEs identified in our assay. In addition, a significant decrease in alternative 5’ end usage was observed in the selected ASEs in comparison to all ASEs (p < 0.0001). The vast majority of the ASEs that are modified upon viral infection were found at a level of one splicing event per gene (Fig 4B). Interestingly, analysis of the correlation between AS and gene expression indicated stable expression of most of the transcripts harboring ASEs that are differentially spliced upon viral infection (Fig 4C). Notable exceptions included GVIN1, an interferon-inducible GTPase, and the apolipoprotein 9a (APOl9A) which are both significantly over-expressed upon viral infection and differentially spliced.

Among the 240 ASEs for which AS was significantly affected upon viral infection, many splicing events we documented affect known protein domains. Table D in S1 Appendix displays the predicted consequences of the differentially spliced transcripts on protein function for 110 transcripts that are differentially spliced upon viral infection. Among the differential ASEs associated with viral infection, at least 10 ASEs resulted in the addition or loss of predicted nuclear localization signals (NLS) including the CDC-Like Kinase 4 (CLK4), the e2F3 transcription factor, and the Influenza Virus NS1A Binding Protein (IVNS1ABP). Other ASEs were associated with key functional domains in proteins such as the loss of the RhoGAP domain in the Rho GTPase activating protein 11A (ARHGAP11a), and the loss of death-effector domains (DED) in the CASP8 And FADD-Like Apoptosis Regulator (CFLAR).

### Proteomic Analysis of Reovirus-Infected Cells

**Fig 5:**
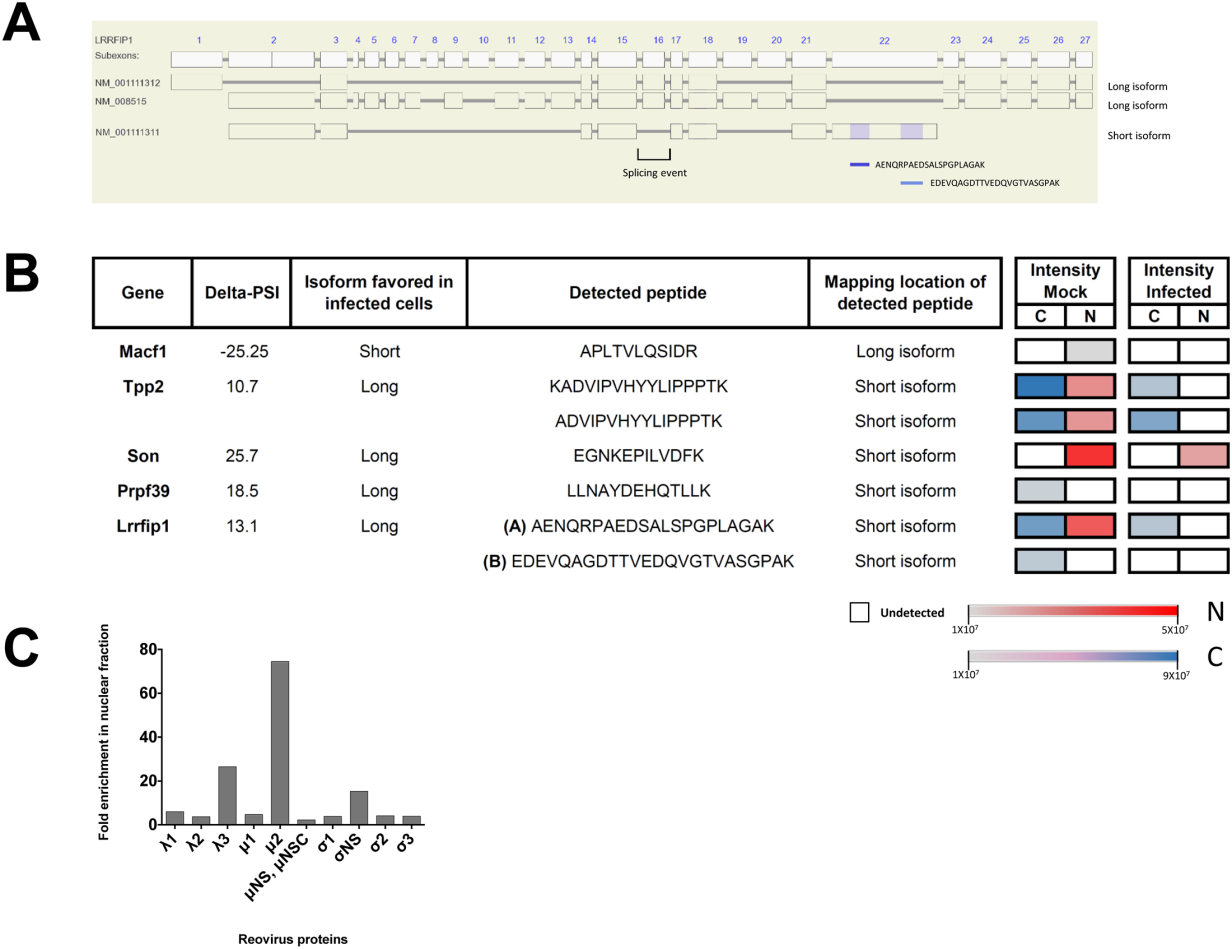
Proteomic analysis of uninfected and reovirus-infected cells. (A) *LRRFIP1* coding exon structure and localization of peptides detected for this gene. Two long isoform transcripts (NM_001111312; NM_008515) and one short isoform transcript (NM_001111311) are also represented. Other short (BC144955; AK044174) and long (BC145642) isoforms analyzed in the RNA-Seq process were omitted. (B) Transcript-specific peptides detected for the long/short transcript. The intensities of peptide detection for both uninfected (mock) and infected cells in the cytoplasm/nucleus fractions are displayed as color gradation (Cytoplasm: grey = weakly detected, blue = strongly detected, white = undetected; Nucleus: grey: lightly detected, red = strongly detected; white = undetected). For Lrrfip1, two peptides (A and B) are presented. (C) Fold enrichment of reovirus proteins in nuclear fraction over the cytoplasmic one. Proteins μ2 (74.5x), λ3 (26.5x) and σNS (15.4x) were found to be above the background level of other viral proteins.

Since we identified changes at the transcriptomic level, we were interested to know if those ASEs have functional consequences at the proteome level during reovirus infection. To assess this question, we infected L929 cells with reovirus and conducted proteomic analysis using LC-MS/MS. Since our informatics analysis predicted that protein localization could be altered in infected cells, nuclear and cytoplasm cell fractionations were realized prior to MS analysis. 42,489 peptides belonging to 4521 genes were detected, of which 100 genes were also previously identified to have altered splicing (100/194: 51.5%). In order to confirm predicted functional changes caused by AS, we used the SpliceVista program which allows visualization and characterization of spliced protein isoforms. The identification of transcripts-specific peptides confirmed that AS modifications in infected cells can modify protein isoform expression from the *MACF1*, *TPP2*, *LRRFIP1*, *PRPF39* and *SON* genes (Fig 5A). For instance, peptide B originating from the LRRFIP1 short transcript isoform was detected by mass spectrometry only in the cytoplasm fraction of the uninfected cells (Fig 5B). A similar pattern was also observed for the peptide originating from the PRPF39 short transcript. The molecular investigation concerning these changes and their impact in alternative splicing and viral-host interaction will be required.

### Conserved RNA Motif and RNA Splicing Factors

The observed differences in the AS landscape of uninfected versus infected cells raised the possibility that AS might be driven by common RNA motifs responding to specific splicing factors. Consequently, the presence of over-represented nucleotide sequences near the splicing regions of the selected ASEs was analyzed. Sequences of the alternative exons and the flanking introns were selected for analysis since they have previously been shown to harbor AS control elements. Notably, we found a significant enrichment of a 41 nt motif in the vicinity of many splice regions of transcripts for which AS was altered upon viral infection (e-value = 1.3e-10^49^) (Fig J in S1 Appendix). The motif was found in 93 of the 240 ASEs that were differentially spliced upon viral infection. Although the exact role of this motif is currently unknown, bioinformatic analyses did not reveal the potential binding of splicing factors to this RNA sequence (data not shown). The AS landscape modification in infected cells could also result from the direct and/or indirect action of specific viral proteins. For instance, a viral protein could be interacting with specific sequences on cellular mRNAs thereby altering normal splicing process. Alternatively, a viral protein could also interact with splicing factors to alter the splicing reaction in the cell nucleus. Previous studies have shown a partial nuclear localization for certain reovirus proteins, namely μ2, σ1s and σ3 [40–47], although other proteins were not necessary examined, especially in the context of infected cells. Interestingly, our proteomics analysis of infected cells revealed nuclear enrichment for μ2, λ3 and σNS (Fig 5C). The potential role of these viral proteins in the virus-induced modification of cellular AS will need to be investigated in future studies.

**Fig 6:**
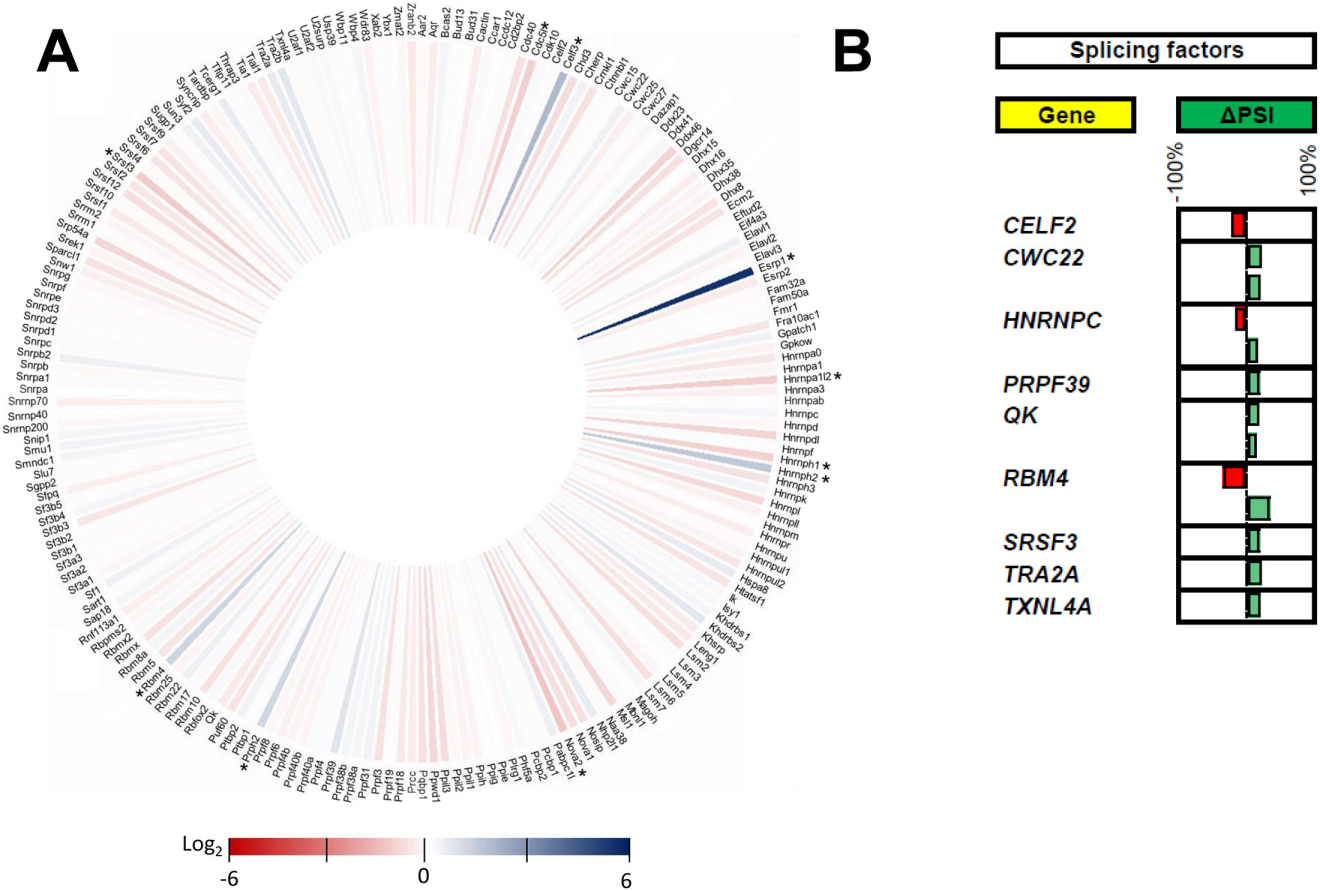
Proteins involved in RNA splicing. (A) Iris graph displaying the expression profile of proteins involved in RNA splicing. Differences in gene expression levels are shown on a logarithmic color scale (Log2). The expression of only 10 proteins involved in splicing was modulated by more than 2-fold upon infection (indicated by an asterisk). (B) Modifications to the splicing profiles of proteins involved in RNA splicing upon viral infection. Nine splicing factors were differentially spliced following viral infection. The changes in PSI values are indicated in red (negative delta PSI) or green (positive delta PSI).

The precise mechanism by which viral infection leads to modifications of the AS landscape in infected cells is currently unknown. Changes in splice site choice frequently arise from modifications in the assembly of the spliceosome or by altering the binding of splicing factors to the RNA transcripts [48]. Although splicing is regulated by an abundant and yet incompletely characterized set of splicing factors, dysregulated expression of individual splicing factors has been shown to frequently result in aberrant splicing [49]. In light of these findings, we therefore monitored both the expression profiles and the modifications in the AS patterns of transcripts encoding spliceosomal proteins and RNA splicing factors. As shown in Fig 6A, the expression of a very limited number of splicing factors and spliceosomal proteins is indeed affected upon viral infection. The expression of only 10 proteins involved in splicing was modulated by more than 2-fold upon infection. Interestingly, the expression of ESRP1, a splicing factor known to regulate diverse types of alternative splicing events [50], was apparently increased by more than 32-fold in infected cells. The ability of reovirus to alter the splicing patterns of transcripts encoding proteins involved in splicing (splicing factors and proteins of the spliceosome) was also investigated. Our study identified 9 splicing factors that were differentially spliced upon viral infection (Fig 6B). One example is the RNA Binding Motif Protein 4 (RBM4), a known modulator of alternative 5’ splice site and exon selection [51]. Modifications to both the expression level and AS of splicing factors likely contribute to the observed modifications in the cellular AS landscape during viral infection.

## Discussion

The present study demonstrated that viral infection can extensively modify the splicing patterns of numerous cellular transcripts involved in gene expression and RNA processing. These splice variants significantly expand proteome diversity and function during viral infection by encoding altered proteins that could influence gene expression and defense homeostasis. These data raise very interesting questions and open new avenues of research for a better understanding of post-transcriptional events during virus infection and possible new targets for the development of antiviral agents.

Many viruses can modify the host cell nuclear functions in order to promote an ideal environment for viral replication. These effects on host cell nuclear functions can be mediated by a variety of mechanisms including the alteration of nuclear architecture [52–55], the interruption of nucleocytoplasmic transport pathways [56–59], and the induction of nuclear herniations [60]. It may come as a surprising observation that infection by a cytoplasmic RNA virus alters the alternative splicing of specific cellular transcripts in the nucleus. In the case of poliovirus, for which alterations to a limited number of transcripts has previously been demonstrated, visible morphological alterations of the nucleus have long been known to occur during viral infection [61]. Such changes are more subtle with reovirus, although “herniations” has been reported in the nucleus of infected cells [42]. Altered DNA synthesis and cell cycle is also observed at least in some cell types and with some reovirus strains [36]. The presence of viral proteins in the nucleus of reovirus-infected cells also supports the idea that this cytoplasmic virus could alter the nuclear splicing machinery. Two likely mechanistic scenarios involve the binding of viral proteins to specific splicing factors thereby hindering their processing activity, and/or the binding of viral proteins to specific sequences on cellular transcripts preventing both binding of splicing factors and subsequent processing.

Dysregulated expression of splicing factors has been shown to be involved in human diseases. For instance, the Serine/Arginine-Rich Splicing Factor 1 (SRSF1) was found to be over-expressed in several tumor types [62], and fibroblasts overexpressing SRSF1 caused tumor formation when injected into mice [63]. Similarly, upregulation of the splicing factor hnRNPH has been shown to drive splicing switches of oncogenic target genes in gliomas [64]. Recently, RBM4, an RNA-binding factor involved in multiple aspects of cellular processes such as AS, was also demonstrated to control cancer-related splicing events affecting cellular processes such as apoptosis, proliferation, and migration [65]. Very few changes in the expression levels and/or splicing patterns of splicing factors were detected in our study upon viral infection. One notable exception is the transcript encoded by *ESRP1* which seemed significantly over-expressed following viral infection (32-fold increase). How the altered expression dynamics of *ESRP1* (and other splicing regulators) contribute to AS homeostasis during virus infection remains to be investigated. Interestingly, a previous study identified ASEs regulated by Esrp1 using RNA silencing technology in a human epithelial cell line [50]. Using such a strategy, the authors identified 148 alternative splicing events in a total of 134 different genes that were regulated by Esrp1. In the present study, the splicing of eight of these Esrp1-regulated transcripts are also found to be differentially spliced upon viral infection (ASPH, JMJD1C, MACF1, NASP, NEDD4L, RBM39, TRA2A, WSB1) suggesting, at least in part, that some of the observed splicing alterations observed upon viral infection could be related to Esrp1 over-expression.

Among the 240 ASEs that were differentially spliced upon viral infection, many have predicted changes in their protein function. Among the latter, the expression of the full-length Mdm2 transcript was found to be significantly reduced and replaced by a shorter form that is predicted to encode a truncated Mdm2 protein deficient in its ability to bind p53. The true relevance of this modification needs to be further confirmed by functional studies. Binding of Mdm2 to p53 has been shown to induce degradation of this key regulator of cell proliferation [66]. Interestingly, stabilization of p53, as is predicted to occur by truncation of the MDM2 protein resulting from alternative splicing modification, was previously shown to increase reovirus oncolytic activity [67]. Indeed, reovirus has been shown to exploit altered Ras signaling pathways in a myriad of cancers [68,69], and this has led to current clinical trials of reovirus as an oncolytic agent [33,34]. Unfortunately, mass spectrometry analysis did not allow the detection of the Mdm2 protein in both uninfected and reovirus-infected cells. Altered splicing pattern of *MDM2* upon viral infection could be another factor contributing to reovirus oncolytic activity and a possible new target to further optimize its therapeutic potential.

Modifications to the global landscape of cellular AS has been thoroughly studied in cancer [10–12]. Various studies are starting to reveal the extent of changes that occur at the splicing level in different types of cancer. Strategies to modulate AS by splice-switching oligonucleotides in order to correct aberrant events, or to induce expression of therapeutic splice variants are being developed [17,20,21]. For instance, the splicing of Bcl-x(L) in cancer cells can be redirected towards the pro-apoptotic variant Bcl-x(S), which has been shown to reduce the tumor load in xenografts of metastatic melanoma [70]. It is therefore tempting to speculate that such a strategy could likely be utilized to limit viral replication. The current identification of extensive changes in the cellular AS landscape during virus-host interactions likely represents a first step toward the development of antiviral agents based on the modulation of AS during viral infections. Molecular tools such as splice-switching oligonucleotides that can specifically alter the proportion of splice variants are also essential to assess the function of these splice variants during viral infection.

## Experimental Procedures

### Cells and Viruses

Murine L929 fibroblasts were obtained from the American Type Culture Collection (ATCC) and were routinely grown in minimal Eagle medium (Wisent) containing 5% fetal bovine serum (FBS Gold, PAA Laboratories). Mammalian orthoreovirus serotype 3 strain Dearing (T3/Human/Ohio/Dearing/55) also originally obtained from ATCC was propagated and titrated by TCID_50_ on L929 fibroblasts, as routinely used in the laboratory [71]. This laboratory stock was recently completely sequenced (NCBI accession numbers KP208804 to KP208813) and rescued by plasmid-based reverse genetics [39]; this virus is referred to as T3D-S. Mutant PL4-12 reovirus was obtained through chemical mutagenesis as described before [38,39].

### Viral Infection

L929 cells were plated at a density of 7X10^4^ cells per square centimeter the day before infection at an MOI of 50TCID_50_ units per cell using standard procedures [71]. Control L929 cells were seeded at the same density and uninfected. Cells were collected 14 hrs post-infection, at which time visible cytopathic effect was still minimal. For RNA-Seq analysis, total RNA was extracted with Trizol^®^ as recommended by the manufacturer (Life Technologies).

### RNA-seq Library Preparation

Messenger RNAs were isolated from 5 ug total RNA using New England Biolabs magnetic mRNA isolation kit (S1550S), as per manufacturer's protocol, and then eluted in 25 μl. Quality and quantity assessments were performed on Agilent Nano Chip (Catalog number 5067–1511); all RIN-Value were equal or above 9.7. The RNA-seq library was then built using Illumina SSV21106 kit from 9 μl isolated mRNA. Library quality was assessed using Agilent DNA HS Chip (Catalog number 5067–4626). Library quantification was performed by qPCR following Illumina Kappa library quantification protocol. WT and P4L-12 reovirus-infected and mock libraries were multiplexed and sequencing was done in triplicate. Pooled libraries were sequenced at 100bp paired-end reads using Illumina HiSeq 2000 at McGill University and Génome Québec Innovation Centre Sequencing Service.

### RNA-seq Data Analysis

Sequence reads were aligned on a transcriptome reference sequence database (UCSCGene MM10) using Bowtie v2 aligner (default parameters) and all valid mapping positions were kept. Associated gene isoforms were quantified in transcript-per-million (TPM) using RSEM for each sequenced sample [72,73]. RSEM uses an Expectation-Maximization (EM) algorithm as its statistical model, allowing reads mapping to multiple transcripts to be also part of the quantification; this huge advantage has lead groups such as the Cancer Genome Atlas (TCGA) to use RSEM in their pipelines. A maximum of two mismatches in the seed (25 bases) was allowed (default parameter). The estimated number of fragments that originate from a specific isoform/gene and the estimated fraction of transcripts corresponding to an isoform/gene are returned by RSEM. Alternative splicing events were automatically detected and quantified using the percent-spliced-in (PSI, Ψ) metric based on long (L) and short (S) forms of all splicing events presents (equation shown below). Briefly, for each splicing events in one given gene (cassette-exon, mutually exclusive exons, alternative 5’ and 3’ splice site, etc), a PSI value was given according to the ratio of the long form on total form present (short form and long form) to characterize inclusion of exon, differential splice-site choice, intron retention, etc. For example, the long form of a cassette-exon would be its inclusion, and short form would be its exclusion from the mature transcript. Events resulting in no change in size (e.g. mutually exclusive exon with the same size) were arbitrary given the long and the short forms.

$$\Psi =\frac{\text{L}}{\text{L}+\text{S}}$$

### Gene Expression Analysis

The gene list was initially filtered to keep only data present in at least two replicates for both virus-infected and mock cells. To ensure higher reproducibility, only genes with expression levels higher than one transcript per million (TPM) in either dataset were conserved. Fold changes in base 2 logarithm were then calculated between infected and mock average TPM. Q-values were calculated to take into account multiple statistical hypothesis testing and results under 0.05 were considered significant.

### Alternative Splicing Analysis

The alternative splicing event (ASE) list was filtered to keep only data with at least two replicates for both virus-infected and mock cells (16,044 genes). Events with a P-value less than 0.05 were conserved (1,732 events). To ensure higher stringency, the ASEs were further filtered with a cutoff Q-value of less than 0.05. From these events, only those with a difference higher than 10% in PSI were considered biologically relevant. The same process was applied to P4L-12 mutant reovirus and mock cells.

### Statistical Analysis

Welch's t-test (Student's t-test with unequal sample sizes and unequal variances) was calculated through the GSL library (http://www.gnu.org/software/gsl/) integrated to Perl system analysis for gene expression and alternative splicing data. Also, false discovery rate was calculated with the Q-value package in R (https://cran.r-project.org/src/contrib/Archive/qvalue/) based on [74]. For all other analysis, Graph Pad Prism version 6.05 was used to run statistics.

### Data Availability

The data discussed in this publication have been deposited in NCBI’s Gene Expression Omnibus [75] and are accessible through GEO Series accession number GSE81017 (https://www.ncbi.nlm.nih.gov/geo/query/acc.cgi?acc=GSE81017).

### Gene Ontology Analysis

The Database for Annotation, Visualization and Integrated Discovery (DAVID) [76] version 6.7 was used and Bonferroni correction was applied to obtained P-values. Reference background was composed of all genes or splicing events analyzed to take into account any bias from the experimental analysis.

### String Networks

Using the STRING database [77] (version 10), genes were submitted for generation of protein-protein interaction network from the *Mus musculus* interactome. High-resolution evidence views were created and saved.

### Consensus Sequence Retrieval

The MEME suite [78] (version 4.10.1) was run against the intron-exon-intron sequence of the selected alternative splicing events, looking for a conserved motif up to 75 nucleotides long. A cutoff of 5 sequences was used to obtain only relevant motifs.

### Functional ASE Prediction

Using the FAST-DB or EASANA suite, the splicing patterns of a gene of interest was visualized. DNA sequences of representative transcripts presenting long and short isoforms were downloaded and translated into proteins using ExPASy translation tool [79]. Counter verification using Genome Browser was done to ensure expression of transcripts and good protein sequence. Predicted proteins were then compared using Multalin (truncation and frameshift event) [80], PFAM and Interpro (loss or appearance of a functional domain) [81,82] and NLS Mapper (loss or gain of nuclear localization signal) [83].

### RT-PCR Validation

Reverse transcription was performed on 2.2 μg total RNA with Transcriptor reverse transcriptase, random hexamers, dNTPs (Roche Diagnostics), and 10 units of RNAse OUT (Invitrogen) following the protocol of the manufacturer in a total volume of 20 μl. All the forward and reverse primers were individually resuspended to 20–100 μM in Tris-EDTA buffer and diluted as a primer pair to 1 μM in RNase DNase-free water (IDT). Quantitative PCR (qPCR) reactions were performed in 10 μl in 96 well plates on a CFX-96 thermocycler (BioRad) with 5 μL of 2X iTaq Universal SYBR Green Supermix (BioRad), 10 ng (3 μl) cDNA, and 200 nM final (2 μl) primer pair solutions. The following cycling conditions were used: 3 min at 95°C; 50 cycles: 15 sec at 95°C, 30 sec at 60°C, 30 sec at 72°C. Relative expression levels were calculated using the qBASE framework. For every PCR run, control reactions performed in the absence of template were performed for each primer pair and these were consistently negative. The amplified products were analyzed by automated chip-based microcapillary electrophoresis on Caliper LC-90 instruments (Caliper LifeSciences). Amplicon sizing and relative quantitation were performed by the manufacturer's software.

### Cell Fractionation

Cells were harvested and pelleted by centrifugation at 1000 RPM for 2 minutes. They were then resuspended in 100 uL magnesium-free PBS; 100 uL of nuclear extraction buffer (40mM Tris-HCl pH 7.5, 20mM MgCl_2_, 4% Triton X-100, 1.28M sucrose) was added with 300 uL of ultrapure water, with all solutions at 4°C. Incubation was carried on ice with occasional agitation by inversion for 20 minutes. Nuclei were retrieved by centrifugation at 5000 RPM, 4°C during 15 minutes; the supernatant was kept as the cytoplasmic fraction. Nuclei were resuspended in 100 uL of low salt buffer (20mM Hepes-KOH pH 7.9, 20mM KCl, 1.5mM MgCl_2_, 20mM EDTA, 0.5mM DTT, 25% glycerol) and 100 uL of KCl 1.2 M was added before harsh agitation. Nuclear lysis was carried for one hour on ice with vigorous vortexing at every 10 minutes. Chromatin was then eliminated by centrifugation at 13 000 RPM for 15 minutes at 4°C and the supernatant represented the nuclear soluble fraction.

### LC-MS/MS Analysis

Proteomic analysis of infected and mock cells was carried out as previously described [84] Briefly, 50 ug of protein from each fraction (nucleus and cytoplasm of infected and mock cells) were reduced in 10 mM DTT and alkylated in 50 mM iodoacetamide. Protein mixture was then separated by one-dimensional SDS-PAGE precast Mini-PROTEAN TGX gel (Bio-Rad). Upon separation, each lane was cut into 4 slices, trypsin-digested and subjected to LC-MS/MS analysis. MaxQuant was used to identify peptide-spectrum match (PSM) against either Uniprot Mus Musculus and Reovirus proteins, or against ENSEMBL *Mus musculus* proteins for SpliceVista analysis.

### SpliceVista Isoform Analysis

*Mus musculus* ENSEMBL ID and gene names were retrieved using BioMart to create the initial database. The code converter.py was modified to permit retrieval of *Mus musculus* gene ID from the ENSEMBL ID database previously created. The SpliceVista program was then run using all default parameters under Linux [85].

## Supporting Information

S1 Appendix Supplemental data (tables and figures). (PDF)

## References

[1] McCormick AL, Mocarski ES Jr. Viral modulation of the host response to infection In: Arvin A, Campadelli-Fiume G, Mocarski E, Moore PS, Roizman B, Whitley R, et al, editors. Human Herpesviruses: Biology, Therapy, and Immunoprophylaxis. Cambridge: Cambridge University Press; 2007 Available: http://www.ncbi.nlm.nih.gov/books/NBK47417/. 21348071

[2] Nagy PD, Pogany J. The dependence of viral RNA replication on co-opted host factors. Nat Rev Microbiol. 2012;10(2):137–49. 10.1038/nrmicro2692 PMC7097227 22183253

[3] Walsh D, Mathews MB, Mohr I. Tinkering with translation: Protein synthesis in virus-infected cells. Cold Spring Harb Perspect Biol. 2013;5(1):a012351 10.1101/cshperspect.a012351 23209131 PMC3579402

[4] Hao L, Sakurai A, Watanabe T, Sorensen E, Nidom CA, Newton MA, et al Drosophila RNAi screen identifies host genes important for influenza virus replication. Nature. 2008;454(7206):890–3. 10.1038/nature07151 18615016 PMC2574945

[5] Kushner DB, Lindenbach BD, Grdzelishvili VZ, Noueiry AO, Paul SM, Ahlquist P. Systematic, genome-wide identification of host genes affecting replication of a positive-strand RNA virus. Proc Natl Acad Sci. 2003;100(26):15764–9. 14671320 10.1073/pnas.2536857100 PMC307642

[6] Black DL. Mechanisms of alternative pre-messenger RNA splicing. Annu Rev Biochem. 2003;72:291–336. 12626338 10.1146/annurev.biochem.72.121801.161720

[7] Pan Q, Shai O, Lee LJ, Frey BJ, Blencowe BJ. Deep surveying of alternative splicing complexity in the human transcriptome by high-throughput sequencing. Nat Genet. 2008;40(12):1413–5. 10.1038/ng.259 18978789

[8] Wang ET, Sandberg R, Luo S, Khrebtukova I, Zhang L, Mayr C, et al Alternative isoform regulation in human tissue transcriptomes. Nature. 2008;456(7221):470–6. 10.1038/nature07509 18978772 PMC2593745

[9] Boise LH, González-García M, Postema CE, Ding L, Lindsten T, Turka LA, et al bcl-x, a bcl-2-related gene that functions as a dominant regulator of apoptotic cell death. Cell. 1993;74(4):597–608. 8358789 10.1016/0092-8674(93)90508-n

[10] Biamonti G, Catillo M, Pignataro D, Montecucco A, Ghigna C. The alternative splicing side of cancer. Semin Cell Dev Biol. 2014;32:30–6. 10.1016/j.semcdb.2014.03.016 24657195

[11] Faustino NA, Cooper TA. Pre-mRNA splicing and human disease. Genes Dev. 2003;17(4):419–37. 12600935 10.1101/gad.1048803

[12] Germann S, Gratadou L, Dutertre M, Auboeuf D, Germann S, Gratadou L, et al Splicing Programs and Cancer. J Nucleic Acids. 2012:e269570. 10.1155/2012/269570 PMC3202119 22132318

[13] Prudencio M, Belzil VV, Batra R, Ross CA, Gendron TF, Pregent LJ, et al Distinct brain transcriptome profiles in C9orf72-associated and sporadic ALS. Nat Neurosci. 2015;18(8):1175–82. 10.1038/nn.4065 26192745 PMC4830686

[14] Wang Z, Burge CB. Splicing regulation: From a parts list of regulatory elements to an integrated splicing code. RNA. 2008;14(5):802–13. 10.1261/rna.876308 18369186 PMC2327353

[15] Matera AG, Wang Z. A day in the life of the spliceosome. Nat Rev Mol Cell Biol. 2014;15(2):108–21. 10.1038/nrm3742 24452469 PMC4060434

[16] Klinck R, Bramard A, Inkel L, Dufresne-Martin G, Gervais-Bird J, Madden R, et al Multiple alternative splicing markers for ovarian cancer. Cancer Res. 2008;68(3):657–63. 10.1158/0008-5472.CAN-07-2580 18245464

[17] Oltean S, Bates DO. Hallmarks of alternative splicing in cancer. Oncogene. 2014;33(46):5311–8. 10.1038/onc.2013.533 24336324

[18] Venables JP, Klinck R, Bramard A, Inkel L, Dufresne-Martin G, Koh C, et al Identification of alternative splicing markers for breast cancer. Cancer Res. 2008;68(22):9525–31. 10.1158/0008-5472.CAN-08-1769 19010929

[19] Venables JP, Klinck R, Koh C, Gervais-Bird J, Bramard A, Inkel L, et al Cancer-associated regulation of alternative splicing. Nat Struct Mol Biol. 2009;16(6):670–6. 10.1038/nsmb.1608 19448617

[20] Alló M, Buggiano V, Fededa JP, Petrillo E, Schor I, de la Mata M, et al Control of alternative splicing through siRNA-mediated transcriptional gene silencing. Nat Struct Mol Biol. 2009;16(7):717–24. 10.1038/nsmb.1620 19543290

[21] Brosseau J-P, Lucier J-F, Lamarche A-A, Shkreta L, Gendron D, Lapointe E, et al Redirecting splicing with bifunctional oligonucleotides. Nucleic Acids Res. 2014;42(6):e40–e40. 10.1093/nar/gkt1287 24375754 PMC3973305

[22] Akusjarvi G. Temporal regulation of adenovirus major late alternative RNA splicing. Front Biosci J Virtual Libr. 2008;13:5006–15. 10.2741/3059 18508565

[23] Johansson C, Schwartz S. Regulation of human papillomavirus gene expression by splicing and polyadenylation. Nat Rev Microbiol. 2013;11(4):239–51. 10.1038/nrmicro2984 23474685

[24] Dowling D, Nasr-Esfahani S, Tan CH, O’Brien K, Howard JL, Jans DA, et al HIV-1 infection induces changes in expression of cellular splicing factors that regulate alternative viral splicing and virus production in macrophages. Retrovirology. 2008;5:18 10.1186/1742-4690-5-18 18241354 PMC2267807

[25] Barnhart MD, Moon SL, Emch AW, Wilusz CJ, Wilusz J. Changes in cellular mRNA stability, splicing and polyadenylation through HuR protein sequestration by a cytoplasmic RNA virus. Cell Rep. 2013;5(4):909–17. 10.1016/j.celrep.2013.10.012 24210824 PMC3849337

[26] Álvarez E, Castelló A, Carrasco L, Izquierdo JM. Poliovirus 2A protease triggers a selective nucleo-cytoplasmic redistribution of splicing factors to regulate alternative pre-mRNA splicing. PLoS ONE. 2013;8(9):e73723 10.1371/journal.pone.0073723 24066065 PMC3774746

[27] Pimienta G, Fok V, Haslip M, Nagy M, Takyar S, Steitz JA. Proteomics and transcriptomics of BJAB cells expressing the Epstein-Barr virus noncoding RNAs EBER1 and EBER2. PLoS ONE. 2015;10(6):e0124638 10.1371/journal.pone.0124638 26121143 PMC4487896

[28] Lindberg A, Kreivi J-P. Splicing inhibition at the level of spliceosome assembly in the presence of herpes simplex virus protein ICP27. Virology. 2002;294(1):189–98. 11886277 10.1006/viro.2001.1301

[29] Sciabica KS. ICP27 interacts with SRPK1 to mediate HSV splicing inhibition by altering SR protein phosphorylation. EMBO J. 2003;22(7):1608–19. 12660167 10.1093/emboj/cdg166 PMC152910

[30] Bryant HE, Wadd SE, Lamond AI, Silverstein SJ, Clements JB. Herpes simplex virus IE63 (ICP27) protein interacts with spliceosome-associated protein 145 and inhibits splicing prior to the first catalytic step. J Virol. 2001;75(9):4376–85. 11287586 10.1128/JVI.75.9.4376-4385.2001 PMC114182

[31] Sandri-Goldin RM, Hibbard MK, Hardwicke MA. The C-terminal repressor region of herpes simplex virus type 1 ICP27 is required for the redistribution of small nuclear ribonucleoprotein particles and splicing factor SC35; however, these alterations are not sufficient to inhibit host cell splicing. J Virol. 1995;69(10):6063–76. 7666511 10.1128/jvi.69.10.6063-6076.1995 PMC189503

[32] Rutkowski AJ, Erhard F, L’Hernault A, Bonfert T, Schilhabel M, Crump C, et al Widespread disruption of host transcription termination in HSV-1 infection. Nat Commun. 2015;6:7126 10.1038/ncomms8126 25989971 PMC4441252

[33] Lee P, Clements D, Helson E, Gujar S. Reovirus in cancer therapy: an evidence-based review. Oncolytic Virotherapy. 2014;3:69–82 10.2147/OV.S51321 27512664 PMC4918368

[34] Chakrabarty R, Tran H, Selvaggi G, Hagerman A, Thompson B, Coffey M. The oncolytic virus, pelareorep, as a novel anticancer agent: a review. Invest New Drugs. 2015;33(3):761–74. 10.1007/s10637-015-0216-8 25693885

[35] Kohl C, Kurth A. Bat reoviruses In: Wang L-F, Cowled C, editors. Bats and Viruses: A New Frontier of Emerging Infectious Diseases. John Wiley & Sons, Inc.; 2015 pp. 203–205.

[36] Tyler KL, Clarke P, DeBiasi RL, Kominsky D, Poggioli GJ. Reoviruses and the host cell. Trends Microbiol. 2001;9(11):560–4. 11825717 10.1016/s0966-842x(01)02103-5

[37] Schurch NJ, Schofield P, Gierliński M, Cole C, Sherstnev A, Singh V, et al How many biological replicates are needed in an RNA-seq experiment and which differential expression tool should you use? RNA. 2016;22(6):839–51. 10.1261/rna.053959.115 27022035 PMC4878611

[38] Rudd P, Lemay G. Correlation between interferon sensitivity of reovirus isolates and ability to discriminate between normal and Ras-transformed cells. J Gen Virol. 2005;86(5):1489–97. 15831962 10.1099/vir.0.80628-0

[39] Sandekian V, Lemay G. A single amino acid substitution in the mRNA capping enzyme λ2 of a mammalian orthoreovirus mutant increases interferon sensitivity. Virology. 2015;483:229–35. 10.1016/j.virol.2015.04.020 25985441 PMC7172830

[40] Bergeron J, Mabrouk T, Garzon S, Lemay G. Characterization of the thermosensitive ts453 reovirus mutant: increased dsRNA binding of sigma 3 protein correlates with interferon resistance. Virology. 1998;246(2):199–210. 9657939 10.1006/viro.1998.9188

[41] Boehme KW, Hammer K, Tollefson WC, Konopka-Anstadt JL, Kobayashi T, Dermody TS. Nonstructural protein σ1s mediates reovirus-induced cell cycle arrest and apoptosis. J Virol. 2013;87(23):12967–79. 10.1128/JVI.02080-13 24067959 PMC3838159

[42] Hoyt CC, Bouchard RJ, Tyler KL. Novel nuclear herniations induced by nuclear localization of a viral protein. J Virol. 2004;78(12):6360–9. 15163729 10.1128/JVI.78.12.6360-6369.2004 PMC416550

[43] Yue Z, Shatkin AJ. Regulated, stable expression and nuclear presence of reovirus double-stranded RNA-binding protein sigma3 in HeLa cells. J Virol. 1996;70(6):3497–501. 8648682 10.1128/jvi.70.6.3497-3501.1996 PMC190223

[44] Zurney J, Kobayashi T, Holm GH, Dermody TS, Sherry B. Reovirus μ2 Protein Inhibits Interferon Signaling through a Novel Mechanism Involving Nuclear Accumulation of Interferon Regulatory Factor 9. J Virol. 2009;83(5):2178–87. 10.1128/JVI.01787-08 19109390 PMC2643726

[45] Ooms LS, Jerome WG, Dermody TS, Chappell JD. Reovirus Replication Protein μ2 Influences Cell Tropism by Promoting Particle Assembly within Viral Inclusions. J Virol. 2012;86(20):10979–87. 22837214 10.1128/JVI.01172-12 PMC3457141

[46] Ooms LS, Kobayashi T, Dermody TS, Chappell JD. A Post-entry Step in the Mammalian Orthoreovirus Replication Cycle Is a Determinant of Cell Tropism. J Biol Chem. 2010;285(53):41604–13. 10.1074/jbc.M110.176255 20978124 PMC3009888

[47] Kobayashi T, Ooms LS, Chappell JD, Dermody TS. Identification of functional domains in reovirus replication proteins μNS and μ2. J Virol. 2009;83(7):2892–906. 10.1128/JVI.01495-08 19176625 PMC2655549

[48] Grosso AR, Martins S, Carmo-Fonseca M. The emerging role of splicing factors in cancer. EMBO Rep. 2008;9(11):1087–93. 10.1038/embor.2008.189 18846105 PMC2581861

[49] Fackenthal JD, Godley LA. Aberrant RNA splicing and its functional consequences in cancer cells. Dis Model Mech. 2008;1(1):37–42. 10.1242/dmm.000331 19048051 PMC2561970

[50] Warzecha CC, Shen S, Xing Y, Carstens RP. The epithelial splicing factors ESRP1 and ESRP2 positively and negatively regulate diverse types of alternative splicing events. RNA Biol. 2009;6(5):546 19829082 10.4161/rna.6.5.9606 PMC2823379

[51] Lu C-C, Chen T-H, Wu J-R, Chen H-H, Yu H-Y, Tarn W-Y. Phylogenetic and molecular characterization of the splicing factor RBM4. PLoS ONE. 2013;8(3):e59092 10.1371/journal.pone.0059092 23527094 PMC3602429

[52] Gustin KE. Effects of poliovirus infection on nucleo-cytoplasmic trafficking and nuclear pore complex composition. EMBO J. 2001;20(1):240–9. 11226174 10.1093/emboj/20.1.240 PMC140206

[53] Gustin KE, Sarnow P. Inhibition of nuclear import and alteration of nuclear pore complex composition by rhinovirus. J Virol. 2002;76(17):8787–96. 12163599 10.1128/JVI.76.17.8787-8796.2002 PMC136411

[54] Muranyi W, Haas J, Wagner M, Krohne G, Koszinowski UH. Cytomegalovirus recruitment of cellular kinases to dissolve the nuclear lamina. Science. 2002;297(5582):854–7. 12161659 10.1126/science.1071506

[55] Scott ES, O’Hare P. Fate of the inner nuclear membrane protein lamin B receptor and nuclear lamins in herpes simplex virus type 1 infection. J Virol. 2001;75(18):8818–30. 11507226 10.1128/JVI.75.18.8818-8830.2001 PMC115126

[56] Enninga J, Levy DE, Blobel G, Fontoura BMA. Role of nucleoporin induction in releasing an mRNA nuclear export block. Science. 2002;295(5559):1523–5. 11809937 10.1126/science.1067861

[57] Fortes P, Beloso A, Ortín J. Influenza virus NS1 protein inhibits pre-mRNA splicing and blocks mRNA nucleocytoplasmic transport. EMBO J. 1994;13(3):704–12. 8313914 10.1002/j.1460-2075.1994.tb06310.x PMC394862

[58] Petersen JM, Her L-S, Varvel V, Lund E, Dahlberg JE. The matrix protein of vesicular stomatitis virus inhibits nucleocytoplasmic transport when it is in the nucleus and associated with nuclear pore complexes. Mol Cell Biol. 2000;20(22):8590–601. 11046154 10.1128/mcb.20.22.8590-8601.2000 PMC102164

[59] Petersen JM, Her L-S, Dahlberg JE. Multiple vesiculoviral matrix proteins inhibit both nuclear export and import. Proc Natl Acad Sci. 2001;98(15):8590–5. 11447272 10.1073/pnas.151240998 PMC37480

[60] de Noronha CMC, Sherman MP, Lin HW, Cavrois MV, Moir RD, Goldman RD, et al Dynamic disruptions in nuclear envelope architecture and integrity induced by HIV-1 Vpr. Science. 2001;294(5544):1105–8. 11691994 10.1126/science.1063957

[61] Reissig M, Howes DW, Melnick JL. Sequence of morphological changes in epithelial cell cultures infected with poliovirus. J Exp Med. 1956;104(3):289–304. 13357686 10.1084/jem.104.3.289 PMC2136577

[62] Anczuków O, Rosenberg AZ, Akerman M, Das S, Zhan L, Karni R, et al The splicing factor SRSF1 regulates apoptosis and proliferation to promote mammary epithelial cell transformation. Nat Struct Mol Biol. 2012;19(2):220–8. 10.1038/nsmb.2207 22245967 PMC3272117

[63] Karni R, de Stanchina E, Lowe SW, Sinha R, Mu D, Krainer AR. The gene encoding the splicing factor SF2/ASF is a proto-oncogene. Nat Struct Mol Biol. 2007;14(3):185–93. 17310252 10.1038/nsmb1209 PMC4595851

[64] Lefave CV, Squatrito M, Vorlova S, Rocco GL, Brennan CW, Holland EC, et al Splicing factor hnRNPH drives an oncogenic splicing switch in gliomas. EMBO J. 2011;30(19):4084–97. 10.1038/emboj.2011.259 21915099 PMC3209773

[65] Wang Y, Chen D, Qian H, Tsai YS, Shao S, Liu Q, et al The splicing factor RBM4 controls apoptosis, proliferation, and migration to suppress tumor progression. Cancer Cell. 2014;26(3):374–89. 10.1016/j.ccr.2014.07.010 25203323 PMC4159621

[66] Okoro DR, Rosso M, Bargonetti J. Splicing Up Mdm2 for Cancer Proteome Diversity. Genes Cancer. 2012;3:311–9. 10.1177/1947601912455323 23150764 PMC3494376

[67] Pan D, Pan L-Z, Hill R, Marcato P, Shmulevitz M, Vassilev LT, et al Stabilisation of p53 enhances reovirus-induced apoptosis and virus spread through p53-dependent NF-κB activation. Br J Cancer. 2011;105(7):1012–22. 10.1038/bjc.2011.325 21863032 PMC3185941

[68] Gong J, Mita MM. Activated Ras signaling pathways and reovirus oncolysis: an update on the mechanism of preferential reovirus replication in cancer cells. Mol Cell Oncol. 2014;4:167. 10.3389/fonc.2014.00167 PMC4071564 25019061

[69] Yang D. RNA Viruses: Host Gene Responses to Infections. World Scientific; 2009 722 p.

[70] Bauman JA, Kole R. Modulation of RNA splicing as a potential treatment for cancer. Bioeng Bugs. 2011;2(3):125–8. 21637003 10.4161/bbug.2.3.15165 PMC3225653

[71] Danis C, Lemay G. Protein synthesis in different cell lines infected with orthoreovirus serotype 3: inhibition of host-cell protein synthesis correlates with accelerated viral multiplication and cell killing. Biochem. Cell Biol. 1993;71(1–2):81–5. 8329180 10.1139/o93-012

[72] Langmead B, Salzberg SL. Fast gapped-read alignment with Bowtie 2. Nat Methods. 2012;9(4):357–9. 10.1038/nmeth.1923 22388286 PMC3322381

[73] Li B, Dewey CN. RSEM: accurate transcript quantification from RNA-Seq data with or without a reference genome. BMC Bioinformatics. 2011;12(1):323. 21816040 10.1186/1471-2105-12-323 PMC3163565

[74] Storey JD, Tibshirani R. Statistical significance for genomewide studies. Proc Natl Acad Sci. 2003;100(16):9440–5. 12883005 10.1073/pnas.1530509100 PMC170937

[75] Edgar R, Domrachev M, Lash AE. Gene Expression Omnibus: NCBI gene expression and hybridization array data repository. Nucleic Acids Res. 2002;30(1):207–10. 11752295 10.1093/nar/30.1.207 PMC99122

[76] Jiao X, Sherman BT, Huang DW, Stephens R, Baseler MW, Lane HC, et al DAVID-WS: a stateful web service to facilitate gene/protein list analysis. Bioinformatics. 2012;28(13):1805–6. 10.1093/bioinformatics/bts251 22543366 PMC3381967

[77] Szklarczyk D, Franceschini A, Wyder S, Forslund K, Heller D, Huerta-Cepas J, et al STRING v10: protein—protein interaction networks, integrated over the tree of life. Nucleic Acids Res. 2015;43(D1):D447–52. 25352553 10.1093/nar/gku1003 PMC4383874

[78] Bailey TL, Boden M, Buske FA, Frith M, Grant CE, Clementi L, et al MEME Suite: tools for motif discovery and searching. Nucleic Acids Res. 2009;37(suppl 2):W202–8. 19458158 10.1093/nar/gkp335 PMC2703892

[79] Artimo P, Jonnalagedda M, Arnold K, Baratin D, Csardi G, de Castro E, et al ExPASy: SIB bioinformatics resource portal. Nucleic Acids Res. 2012;40(W1):W597–603. 22661580 10.1093/nar/gks400 PMC3394269

[80] Corpet F. Multiple sequence alignment with hierarchical clustering. Nucleic Acids Res. 1988;16(22):10881–90. 2849754 10.1093/nar/16.22.10881 PMC338945

[81] Finn RD, Bateman A, Clements J, Coggill P, Eberhardt RY, Eddy SR, et al Pfam: the protein families database. Nucleic Acids Res. 2014;42(D1):D222–30. 24288371 10.1093/nar/gkt1223 PMC3965110

[82] Mitchell A, Chang H-Y, Daugherty L, Fraser M, Hunter S, Lopez R, et al The InterPro protein families database: the classification resource after 15 years. Nucleic Acids Res. 2015;43(D1):D213–21. 25428371 10.1093/nar/gku1243 PMC4383996

[83] Kosugi S, Hasebe M, Tomita M, Yanagawa H. Systematic identification of cell cycle-dependent yeast nucleocytoplasmic shuttling proteins by prediction of composite motifs. Proc Natl Acad Sci U S A. 2009;106(25):10171–6. 10.1073/pnas.0900604106 19520826 PMC2695404

[84] Drissi R, Dubois M-L, Douziech M, Boisvert F-M. Quantitative proteomics reveals dynamic interactions of the minichromosome maintenance complex (MCM) in the cellular response to etoposide induced DNA damage. Mol Cell Proteomics. 2015;14(7):2002–13. 10.1074/mcp.M115.048991 25963833 PMC4587322

[85] Zhu Y, Hultin-Rosenberg L, Forshed J, Branca RMM, Orre LM, Lehtiö J. SpliceVista, a tool for splice variant identification and visualization in shotgun proteomics data. Mol Cell Proteomics. 2014;13(6):1552–62. 10.1074/mcp.M113.031203 24692640 PMC4047474

