# Supplementary material for: Global Profiling of the Cellular Alternative RNA Splicing Landscape during Virus-Host Interactions
Source: PLoS One. 2016 Sep 6;11(9):e0161914. doi: 10.1371/journal.pone.0161914 (PMC5012649; doi:10.1371/journal.pone.0161914)
Supplement: S1 Appendix — (PDF) [file pone.0161914.s001.pdf]

Supplemental Data

Supplemental Tables and Figures

Global Profiling of the Cellular Alternative RNA Splicing Landscape During Virus-host Interactions

Simon Boudreault[1], Camille Martenon-Brodeur[1], Marie Caron[1], Jean-Michel Garant[1], Marie-Pier Tremblay[1], Victoria E. S. Armero[1], Mathieu Durand[2], Elvy Lapointe[2], Philippe Thibault[2], Maude Tremblay-Létourneau[1], Jean-Pierre Perreault[1], Michelle S. Scott[1], Guy Lemay[3], Martin Bisaillon[1,*]

Table A. RNA-sequencing data		
**Sample**	**Number of bases**	**HiSeq Reads**
Mock 1	9 270 066 200	46 350 331
Mock 2	9 400 946 800	47 004 734
Mock 3	7 880 753 600	39 403 768
Reovirus 1	10 499 367 400	52 496 837
Reovirus 2	10 352 922 800	51 764 614
Reovirus 3	8 850 997 600	44 254 988

RNA-seq was performed on uninfected (mock-infected) cells, and cells infected with reovirus (14 hours post-infection). Sequencing was done in triplicate.

Table B. Distribution of ΔPSI values in the 240 differentially spliced ASEs

ΔPSI	Number of ASEs modified
[-80,-70[	1
[-70,-60[	0
[-60,-50[	4
[-50,-40[	4
[-40,-30[	15
[-30,-20[	35
[-20,-10[	60
]10,20]	64
]20,30]	39
]30,40]	11
]40,50]	3
]50,60]	2
]60,70]	2

Table C: Sequencing results (Sanger) for splicing junctions confirmed by RT-PCR

Gene	ASE Form	Splicing junction	Predicted sequence	Sequencing results
Il34 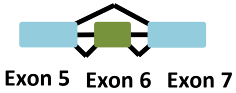	Short	5-7	CAGCGGAGCCT CATGGATGTGG AGATTGGC	CAGCGGAGCCT CATGGATGTGG AGATTGGC
	Long	5-6	NA	NA
	Long	6-7	NA	NA
hnRNPA2B1 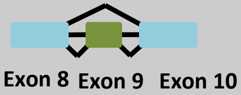	Short	8-10	GAGGAGGACCT GGAGGAAATTA TGGAAGTG	GAGGAGGACCT GGAGGAAATTA TGGAAGTG
	Long	8-9	GAGGAGGACCT GGAGGTGGCAA TTTTGGAG	GAGGAGGACCT GGAGGTGGCAA TTTTGGAG
	Long	9-10	ACAACTATGGA GGAGGAAATTA TGGAAGTG	ACAACTATGGA GGAGGAAATTA TGGAAGTG

NA: Splicing junction expressed at levels too low to allow sequencing

Table D. Bioinformatic prediction of functional changes caused by some identified ASEs

Gene	Delta-PSI	Event Type	Full name	Predicted residue variation caused by alternative splicing modification (ΔAA)	Predicted functional consequence
**Abi1**	-50.8	exon-cassette+alternate-3p+multiple-exon-cassette	abl-interactor 1	-29	Unknown
**Adar**	-25.1	alternate-5p	adenosine deaminase, RNA-specific	-26	Unknown
**Agfg1**	-27.6	alternate-3p	ArfGAP with FG repeats 1	-19	Unknown
**Apol9a**	22.9	alternate-5p	apolipoprotein L 9a	0	No predicted functional change
**Arhgap11a**	-42.9	exon-cassette+alternate-3p+intron-retention	Rho GTPase activating protein 11A	-515	NLS and RhoGAP domain loss in short isoform
**Arhgap11a**	16.7	exon-cassette+alternate-3p+intron-retention	Rho GTPase activating protein 11A	-489	Premature stop codon introduced by intron retention in long isoform
**Asph**	13.8	exon-cassette	aspartate-beta-hydroxylase	83	Unknown
**Atat1**	13.8	exon-cassette	alpha tubulin acetyltransferase 1	23	Unknown
**Atxn7l1**	-22.4	exon-cassette	ataxin 7-like 1	-51	Unknown
**Bnip2**	-18.3	exon-cassette	BCL2/adenovirus E1B interacting protein 2	-6	Unknown
**Bora**	37.5	exon-cassette	bora, aurora kinase A activator	-251	Premature stop codon in long isoform
**Bptf**	-22.9	alternate-5p	bromodomain PHD finger transcription factor	-281	Unknown
**Calu**	13.3	mutually-exclusive-exon	Calumenin	0	Unknown
**Cbwd1**	-23.1	alternate-5p	COBW domain containing 1	-121	CobW/HypB/UreG domain truncated in short isoform
**Ccdc136**	-10.2	exon-cassette	coiled-coil domain containing 136	-47	Unknown
**Ccdc15**	14.8	alternate-5p	coiled-coil domain containing 15	116	Unknown
**Cdkn2aip**	-18.2	alternate-5p	CDKN2A interacting protein	-444	Premature stop codon inducing a NLS loss in short isoform
**Celf1**	11.2	alternate-3p	CUGBP, Elav-like family member 1	1	Unknown

Table D

Gene	Delta-PSI	Event Type	Full name	Predicted residue variation caused by alternative splicing modification (ΔAA)	Predicted functional consequence
**Cflar**	-14.8	exon-cassette	CASP8 and FADD-like apoptosis regulator	-186	Loss of the two DED domains in short isoform
**Cflar**	27.6	exon-cassette	CASP8 and FADD-like apoptosis regulator	-186	Loss of the two DED domains in long isoform
**Clip1**	-18.9	alternate-5p	CAP-GLY domain containing linker protein 1	-76	Unknown
**Clk4**	11.9	exon-cassette+intron-retention	CDC like kinase 4	180	Gain of 10 NLS and full-length Pkinase domain in long isoform
**Cwc22**	21.5	alternate-5p	CWC22 spliceosome-associated protein homolog (S, cerevisiae)	-6	Unknown
**Cwc22**	19.9	exon-cassette	CWC22 spliceosome-associated protein homolog (S, cerevisiae)	6	Unknown
**E2f3**	-20.9	alternate-3p	E2F transcription factor 3	-6	Additionnal NLS in short isoform
**Edc3**	-26.5	exon-cassette	enhancer of mRNA decapping 3 homolog (S, cerevisiae)	-183	Loss of Edc3-linker domain, LSM14 domain and truncation of YjwF_N domain in short isoform
**Eif4a2**	-11.4	exon-cassette	eukaryotic translation initiation factor 4A2	-95	Truncation of DEAD box helicase domain in short isoform
**Eif4h**	-16.7	exon-cassette	eukaryotic translation initiation factor 4H	-20	Unknown
**Erbb2ip**	-30.0	exon-cassette+alternate-3p+multiple-exon-cassette+mutually-exclusive-exon	Erbb2 interacting protein	-39	Unknown
**Erbb2ip**	23.3	exon-cassette+alternate-3p+multiple-exon-cassette+mutually-exclusive-exon	Erbb2 interacting protein	-384	Premature stop codon, C-Term PDZ domain loss in short isoform
**Fam179b**	-30.2	exon-cassette	family with sequence similarity 179, member B	-50	Unknown

Table D (cont'd)

Gene	Delta-PSI	Event Type	Full name	Predicted residue variation caused by alternative splicing modification (ΔAA)	Predicted functional consequence
**Flcn**	35.8	alternate-3p	folliculin	-129	Premature stop codon, resulting in Folliculin domain truncation (174AA, C-term)
**Foxk2**	-29.7	exon-cassette	forkhead box K2	-401	Unknown
**G2e3**	-53.0	exon-cassette+alternate-3p+multiple-exon-cassette	G2/M-phase specific E3 ubiquitin ligase	116	Unknown
**Gdap2**	15.1	alternate-3p	ganglioside-induced differentiation-associated-protein 2	256	Complete CRAL and TRIO domains in long isoform
**Gen1**	-28.7	exon-cassette	Gen homolog 1, endonuclease (Drosophila)	-10	Earlier start codon in long isoform; Short isoform loses its XPG_N domain in N-term
**Gls**	11.3	alternate-3p	glutaminase	-376	Unknown
**Gpbp1**	25.6	exon-cassette	GC-rich promoter binding protein 1	20	Unknown
**Hmga1**	15.6	exon-cassette+alternate-5p+intron-retention	high mobility group AT-hook 1	0	5'UTR sequence change
**Hnrnpa2b1**	-13.6	exon-cassette	heterogeneous nuclear ribonucleoprotein A2/B1	-40	Additionnal HnRNPA1 domain in N-term of short isoform
**Il34**	58.9	exon-cassette	interleukin 34	-16	Frameshift introducing a premature stop codon in exon 8; truncation of Il34 domain
**Ilf3**	13.2	exon-cassette	interleukin enhancer binding factor 3	13	Unknown
**Iqce**	17.6	exon-cassette	IQ motif containing E	17	Unknown
**Iqce**	-19.2	exon-cassette	IQ motif containing E	-17	Unknown
**Ivns1abp**	30.8	exon-cassette+alternate-3p	influenza virus NS1A binding protein	-281	NLS and BTB domains loss in long isoform

Table D (cont’d)

Gene	Delta-PSI	Event Type	Full name	Predicted residue variation caused by alternative splicing modification (ΔAA)	Predicted functional consequence
**Kat5**	28.7	exon-cassette	K(lysine) acetyltransferase 5	52	Long isoform have two NLS instead of only one in the short isoform
**Kat6a**	-13.6	exon-cassette	K(lysine) acetyltransferase 6A	130	Unknown
**Kmt2e**	10.8	exon-cassette	lysine (K)-specific methyltransferase 2E	-1602	Loss of 6 NLS and SET and PHD domains in long isoform
**Lrrfip1**	13.1	exon-cassette	leucine rich repeat (in FLII) interacting protein 1	24	Truncation in double-stranded RNA binding protein DUF2051 in long isoform
**Macf1**	-25.2	multiple-exon-cassette	microtubule-actin crosslinking factor 1	-2027	Loss of all 9 Plectin repeat domains in short isoform
**Matr3**	12.3	alternate-5p	matrin 3	0	No predicted functional change
**Max**	11.3	exon-cassette	Max protein	-122	NLS and Helix-Loop-Helix domain loss in long isoform
**Mbnl2**	24.1	exon-cassette	muscleblind-like 2	18	Unknown
**Mdm2**	-58.8	exon-cassette+alternate-5p	transformed mouse 3T3 cell double minute 2	-197	SWIB domain in N-Term loss in short isoform
**Mon2**	38.3	exon-cassette	MON2 homolog (yeast)	7	6 residue gap in Mon2 domain in short isoform
**Morn1**	-16.3	alternate-5p	MORN repeat containing 1	-245	Loss of 4 MORN domain out of 8 in short isoform
**Msh4**	-38.7	alternate-5p	mutS homolog 4 (E, coli)	404	Premature stop codon in long isoform
**Mtif3**	25.0	exon-cassette+alternate-5p	mitochondrial translational initiation factor 3	0	Unknown
**Nabp1**	41.4	alternate-5p+intron-retention	nucleic acid binding protein 1	-91	Unknown
**Naip2**	21.8	exon-cassette	NLR family, apoptosis inhibitory protein 2	56	Unknown

Table D (cont'd)

Gene	Delta-PSI	Event Type	Full name	Predicted residue variation caused by alternative splicing modification (ΔAA)	Predicted functional consequence
**Nasp**	-16.5	exon-cassette	nuclear autoantigenic sperm protein (histone-binding)	-325	Unknown
**Nde1**	-16.6	alternate-3p	nuclear distribution gene E homolog 1 (A nidulans)	27	Unknown
**Nek1**	26.9	exon-cassette	NIMA (never in mitosis gene a)-related expressed kinase 1	29	Unknown
**Neurl4**	-19.2	exon-cassette	neuralized homolog 4 (Drosophila)	-22	Unknown
**Nfx1**	28.3	alternate-3p	nuclear transcription factor, X-box binding 1	-14	Unknown
**Odf2l**	-16.0	exon-cassette	outer dense fiber of sperm tails 2-like	-96	Unknown
**Opa1**	-14.5	exon-cassette	optic atrophy 1	-37	Unknown
**Pan3**	17.4	exon-cassette	PAN3 polyA specific ribonuclease subunit homolog (S, cerevisiae)	258	Unknown
**Pcgf5**	15.5	exon-cassette	polycomb group ring finger 5	20	C-term RAWUL domain truncated in long isoform
**Phactr4**	-16.8	exon-cassette	phosphatase and actin regulator 4	-27	RPEL repeat domain loss in short isoform
**Piga**	-33.8	alternate-5p	phosphatidylinositol glycan anchor biosynthesis, class A	-316	PIGA domain loss in short isoform
**Pitpnb**	-21.8	exon-cassette	phosphatidylinositol transfer protein, beta	-1	Unknown
**Pnpla7**	69.1	alternate-5p	patatin-like phospholipase domain containing 7	-886, -52, -454 or +380	Unknown
**Polg2**	-13.5	alternate-5p	polymerase (DNA directed), gamma 2, accessory subunit	-44	Unknown
**Ppfibp1**	-16.1	exon-cassette+intron-retention	PTPRF interacting protein, binding protein 1 (liprin beta 1)	-11	Unknown

Table D (cont'd)

Gene	Delta-PSI	Event Type	Full name	Predicted residue variation caused by alternative splicing modification (ΔAA)	Predicted functional consequence
**Ppp1r12a**	10.2	exon-cassette	protein phosphatase 1, regulatory (inhibitor) subunit 12A	422	Unknown
**Pqlc1**	50.9	exon-cassette	PQ loop repeat containing 1	18	Unknown
**Prpf39**	18.5	exon-cassette+multiple-exon-cassette	PRP39 pre-mRNA processing factor 39 homolog (yeast)	-201, -239	Loss of part of Tetratricopeptide-like helical domain and HAT reapeats in both C- and N-term
**Pum2**	-11.2	exon-cassette	pumilio RNA-binding family member 2	-79	Unknown
**Ranbp3**	-27.0	exon-cassette+multiple-exon-cassette	RAN binding protein 3	331	NLS loss and full RanBP1 domain in long isoform
**Rfx5**	34.7	exon-cassette+alternate-5p	regulatory factor X, 5 (influences HLA class II expression)	185	NLS gain and full-length RFX5 DNA-binding domain in long isoform
**Rnf135**	-31.1	exon-cassette	ring finger protein 135	-216	Zinc finger of C3HC4-type domain loss in short isoform
**Rnps1**	-25.7	exon-cassette+multiple-exon-cassette	ribonucleic acid binding protein S1	-23	Unknown
**Rnps1**	-14.6	exon-cassette+multiple-exon-cassette	ribonucleic acid binding protein S1	-23	Unknown
**Scaf11**	-11.6	alternate-5p	SR-related CTD-associated factor 11	-16	Unknown
**Senp1**	22.9	exon-cassette	SUMO1/sentrin specific peptidase 1	-142	Unknown
**Sfswap**	-24.8	alternate-5p	splicing factor, suppressor of white-apricot family	-724	47 AA truncation in Alternative splicing regulator domain and 2 Surp module domain loss in short isoform
**Skil**	-14.2	alternate-5p	SKI-like	-35	Unknown

Table D (cont’d)

Gene	Delta-PSI	Event Type	Full name	Predicted residue variation caused by alternative splicing modification (ΔAA)	Predicted functional consequence
**Slc23a2**	-12.2	alternate-3p	solute carrier family 23 (nucleobase transporters), member 2	-308	208 AA loss in the N-term of Xan_ur_permease domain in short isoform
**Son**	25.7	exon-cassette+alternate-5p	Son DNA binding protein	-2198	Premature stop codon in long isoform, introducing 4 NLS and G-patch, Arginine/Serine-Rich protein 1 and double-stranded RNA binding domain loss
**Spef2**	18.4	exon-cassette	sperm flagellar 2	923	Unknown
**Spopl**	27.8	exon-cassette	speckle-type POZ protein-like	56	Unknown
**Stx3**	39.5	exon-cassette+mutually-exclusive-exon	syntaxin 3	97	75 AA truncation of Syntaxin domain in long isoform
**Tbp**	-44.3	exon-cassette	TATA box binding protein	-56	TBP domain loss and truncation of first TBP domain's end in short isoform
**Tcf12**	30.7	exon-cassette+intron-retention	transcription factor 12	24	Unknown
**Tmem132a**	66.1	alternate-3p	transmembrane protein 132A	29	Unknown
**Tmem168**	-32.5	exon-cassette	transmembrane protein 168	-384	Unknown
**Tmem19**	-15.0	alternate-5p	transmembrane protein 19	12	Full lenght DUF92 integral membrane domain in long isoform
**Tmpo**	-38.3	exon-cassette+multiple-exon-cassette	thymopoietin	-40	Unknown
**Tmpo**	-11.5	exon-cassette+multiple-exon-cassette	thymopoietin	-72	Unknown
**Tpp2**	10.7	exon-cassette	tripeptidyl peptidase II	-130	Unknown
**Tra2a**	21.2	exon-cassette	transformer 2 alpha homolog (Drosophila)	97	Full-length RNA recognition motif in long isoform

Table D (cont’d)

Gene	Delta-PSI	Event Type	Full name	Predicted residue variation caused by alternative splicing modification (ΔAA)	Predicted functional consequence
**Trappc13**	-29.9	exon-cassette	trafficking protein particle complex 13	-6	DUF974 domain truncation of 6 AA in short isoform
**Ubap1**	-21.4	exon-cassette	ubiquitin-associated protein 1	-61	Unknown
**Ubr3**	11.3	alternate-3p	ubiquitin protein ligase E3 component n-recognin 3	4	Unknown
**Upf1**	-23.4	alternate-5p	UPF1 regulator of nonsense transcripts homolog (yeast)	-11	Unknown
**Wsb1**	19.9	alternate-3p	WD repeat and SOCS box-containing 1	-177	Premature stop codon in long isoform, resulting in a truncated protein containing only a WD, G-beta domain instead of 4 WD, G-beta domain and a SOCS box domain
**Xdh**	-25.6	alternate-5p	xanthine dehydrogenase	-599	NLS appearance in short isoform
**Zfp207**	-12.5	exon-cassette	zinc finger protein 207	-6	Unknown
**Zfp788**	-32.4	exon-cassette+alternate-3p+multiple-exon-cassette	zinc finger protein 788	-532	Unknown
**Zmynd11**	-17.1	exon-cassette	zinc finger, MYND domain containing 11	-54	Unknown
**Zyx**	13.8	exon-cassette	zyxin	31	Unknown
**1700057G04Rik**	-31.1	exon-cassette	RIKEN cDNA 1700057G04 gene	-76	Truncation in Scramblase domain in short isoform
**2310057M21Rik**	-15.9	alternate-5p	RIKEN cDNA 2310057M21 gene	77	Unknown
**4631405J19Rik**	-45.7	alternate-3p	RIKEN cDNA 4631405J19 gene	-100	Unknown
**4930511M06Rik**	-23.6	exon-cassette	RIKEN cDNA 4930511M06 gene	NA	Non-coding RNA

Table D (cont'd)

Figure A. Detection of reovirus genes in infected cells by qRT-PCR.

(A) Total RNA was extracted from both uninfected and infected cells (14 h post-infection). Viral infection was confirmed by qRT-PCR using specific primers for three viral genes (M1, S1, L1). A control reaction without template (NTC: no template control) was also performed. All three viral genes were detected in the infected cells samples but were undetectable in the uninfected (mock) samples (absence of amplification or nonspecific amplification). Cq: quantification cycle.

(B) Primers used for qPCR detection.

A

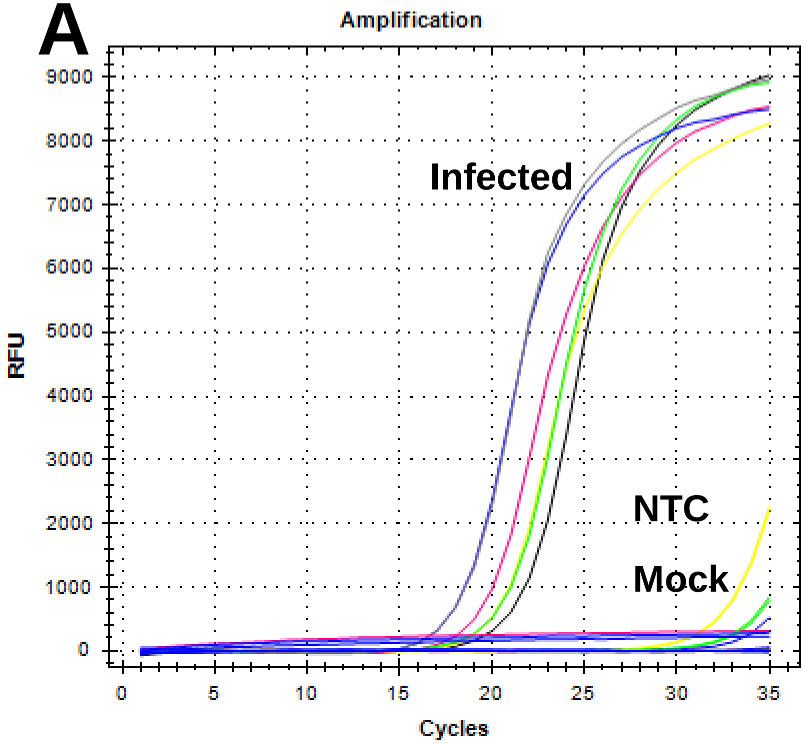

qRT-PCR data on amplification of viral genes in uninfected (mock) and infected cells

Orthoreovirus Primer	Cq Mock	Cq infected	Cq NTC	Color
L1_1	Undetected	20.52	Undetected	Black
L1_2	Undetected	19.22	Undetected	Yellow
M1_1	Undetected	18.57	Undetected	Pink
M1_2	Undetected	19.68	Undetected	Green
S1_1	Undetected	16.93	Undetected	Blue
S1_2	Undetected	17.00	Undetected	Gray

B

Primers design for qRT-PCR amplification of reovirus genes

Name	Primer forward	Sequence	Primer reverse	Sequence
L1_1	orthoreovirus_t3d_seg_L1.F1	GGCAAAGACGGTGTCGGGTC	orthoreovirus_t3d_seg_L1.R1	TGCGTCCGCTTCTGACTCCT
L1_2	orthoreovirus_t3d_seg_L1.F2	CTAGGGGTGGATCTGGCGCA	orthoreovirus_t3d_seg_L1.R2	TCGCTAATTGTGCCGCCTGG
M1_1	orthoreovirus_t3d_seg_M1.F1	AGCGCGCAGCCTAAATGGTT	orthoreovirus_t3d_seg_M1.R1	ACGTAGATGCCGGGTCTGCT
M1_2	orthoreovirus_t3d_seg_M1.F2	AGATGAGGCTTTCGTGCGGC	orthoreovirus_t3d_seg_M1.R2	GCATGTCACGGATCACGCCA
S1_1	orthoreovirus_t3d_seg_S1.F1	GGTCGGATGGATCCTCGCCT	orthoreovirus_t3d_seg_S1.R1	TCGAGTCCCTGGGTGATCCG
S1_2	orthoreovirus_t3d_seg_S1.F2	CCTCCGGATCACCCAGGGAC	orthoreovirus_t3d_seg_S1.R2	TGTCCCACTCGAGCACCCAA

Figure A

Figure B. Heatmap representation of gene expression of total cellular genes in both infected and uninfected (mock) cells.

RNA sequencing was done in triplicate for each condition. The map represents the number of transcripts sequences (TPM: transcripts per million) in a color-coded logarithmic scale. Blue and green indicates high levels of gene expression while yellow indicates low levels of gene expression.

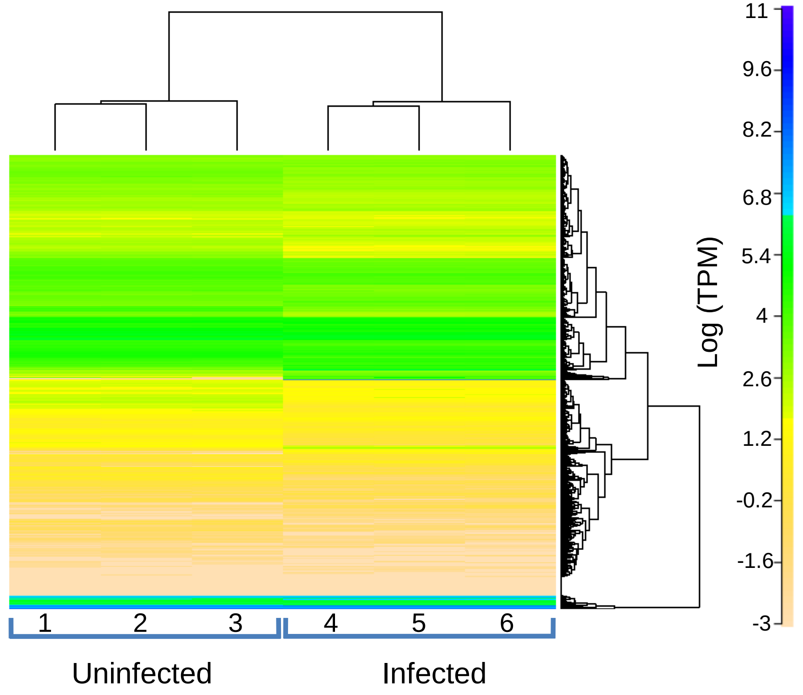

11
9.6
8.2
6.8
5.4
4
2.6
1.2
-0.2
-1.6
-3
Log (TPM)
1
2
3
4
5
6
Uninfected
Infected

Figure B

Figure C. List of the 570 genes for which the expression was the most significantly modified following viral infection.

The list of genes is shown with the corresponding expression profile of each gene. The level of gene expression is presented on a logarithmic scale (Log_2)

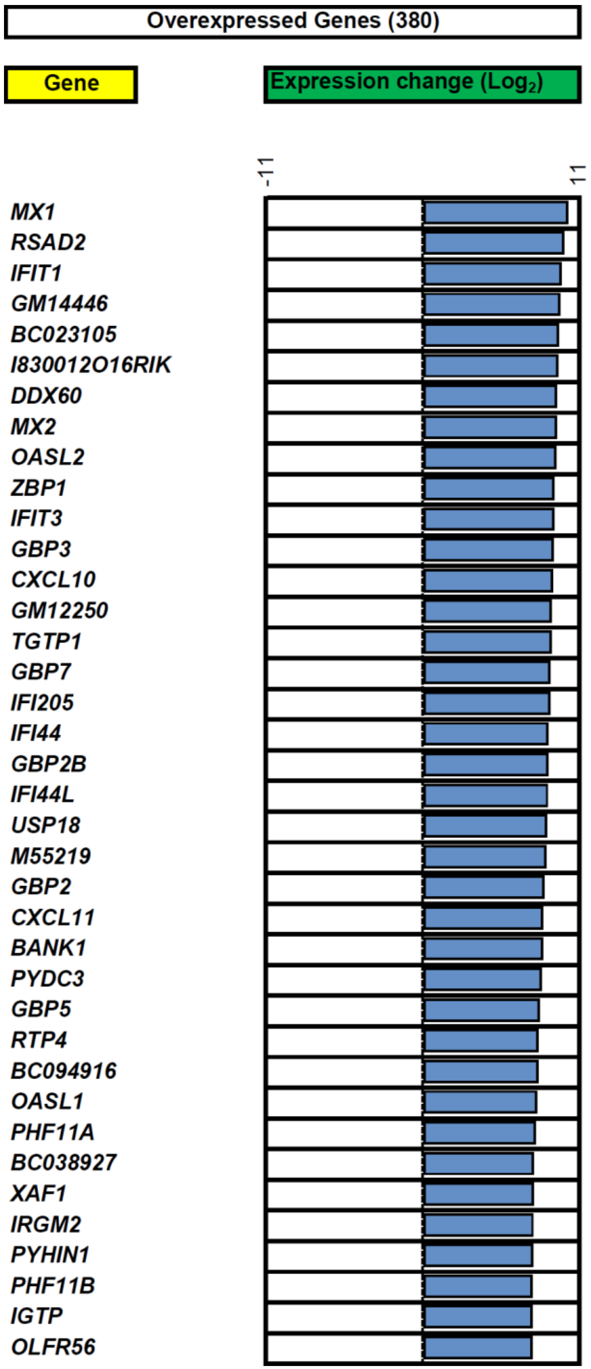
Overexpressed Genes (380)
Gene
Expression change (Log2)
-11
11
MX1
RSAD2
IFIT1
GM14446
BC023105
I830012O16RIK
DDX60
MX2
OASL2
ZBP1
IFIT3
GBP3
CXCL10
GM12250
TGTP1
GBP7
IFI205
IFI44
GBP2B
IFI44L
USP18
M55219
GBP2
CXCL11
BANK1
PYDC3
GBP5
RTP4
BC094916
OASL1
PHF11A
BC038927
XAF1
IRGM2
PYHIN1
PHF11B
IGTP
OLFR56

Figure C

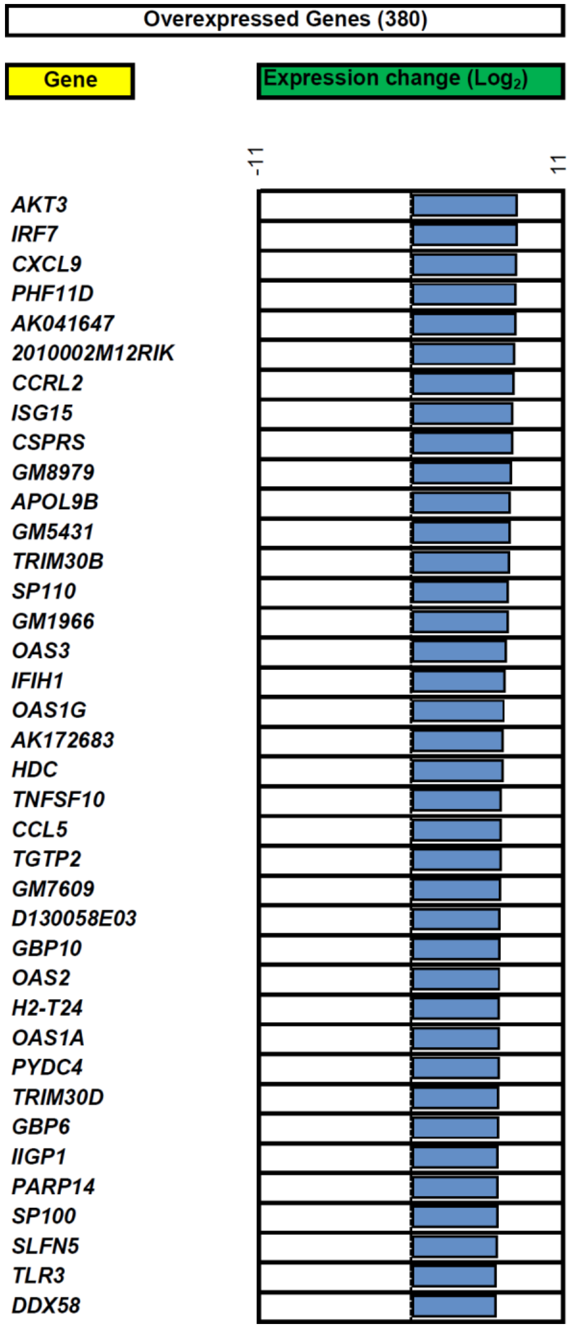

Figure C (cont'd)

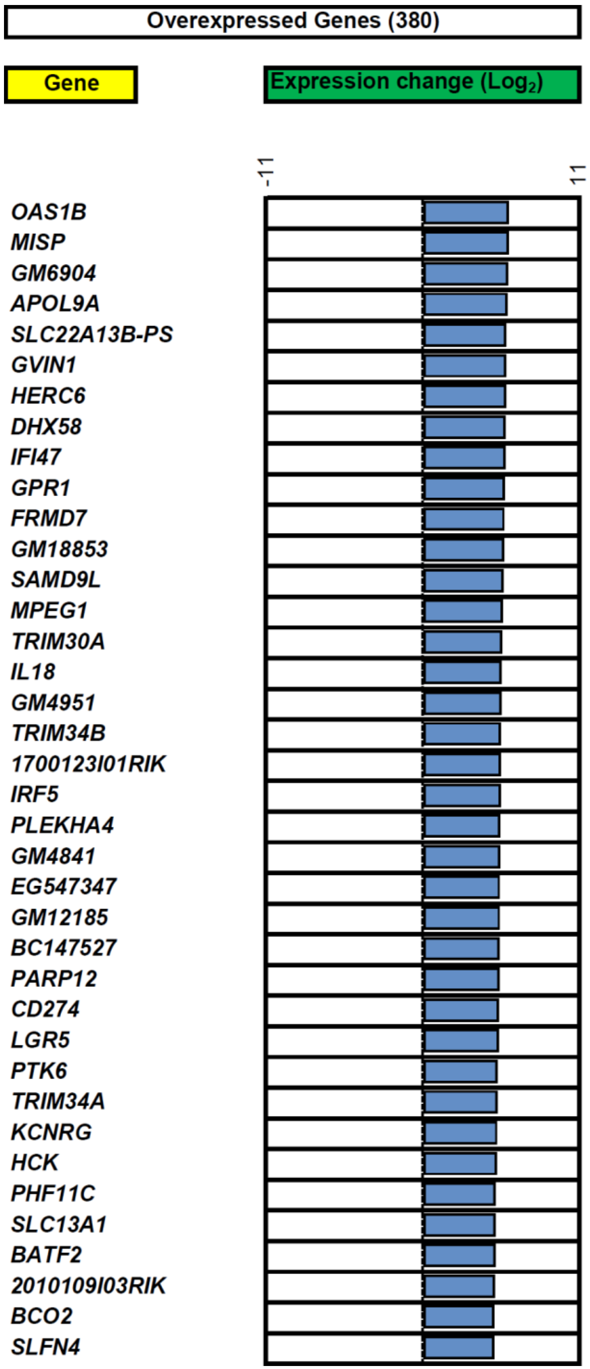

Figure C (cont'd)

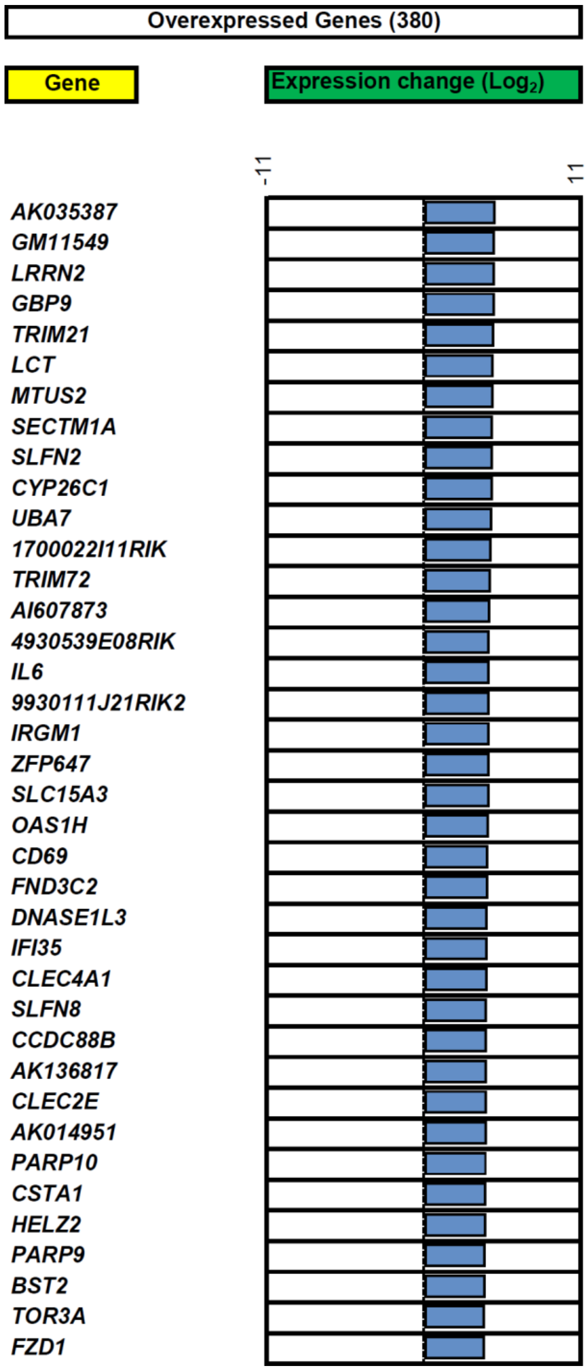

Figure C (cont'd)

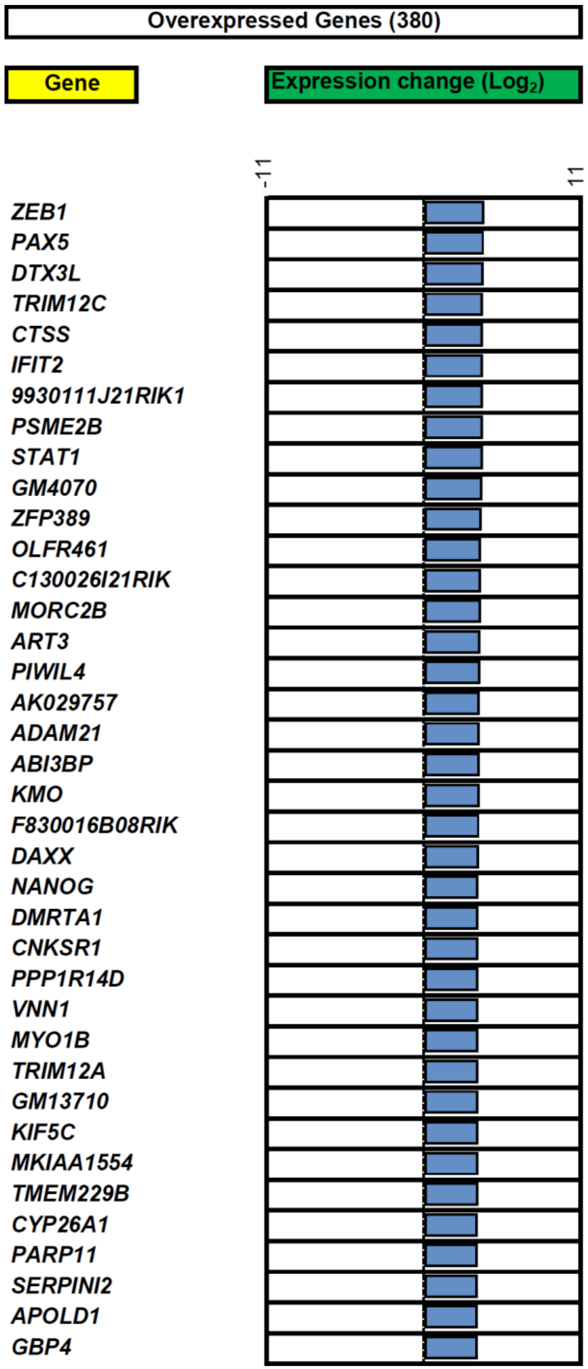

Figure C (cont’d)

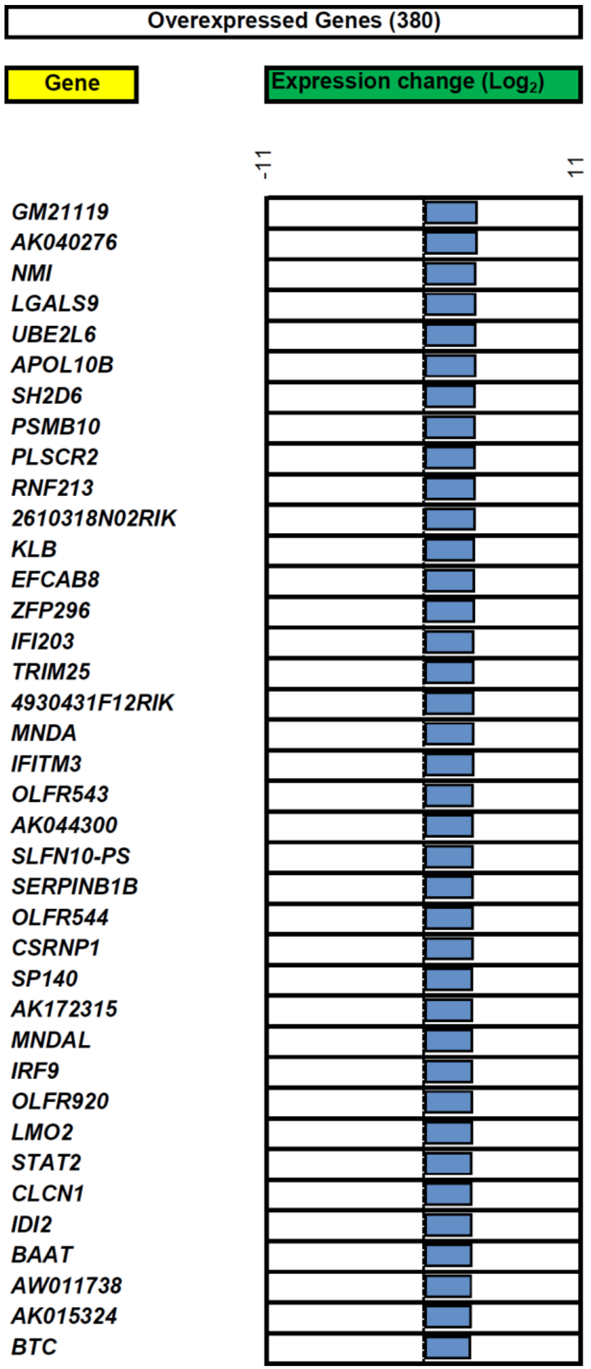

Figure C (cont’d)

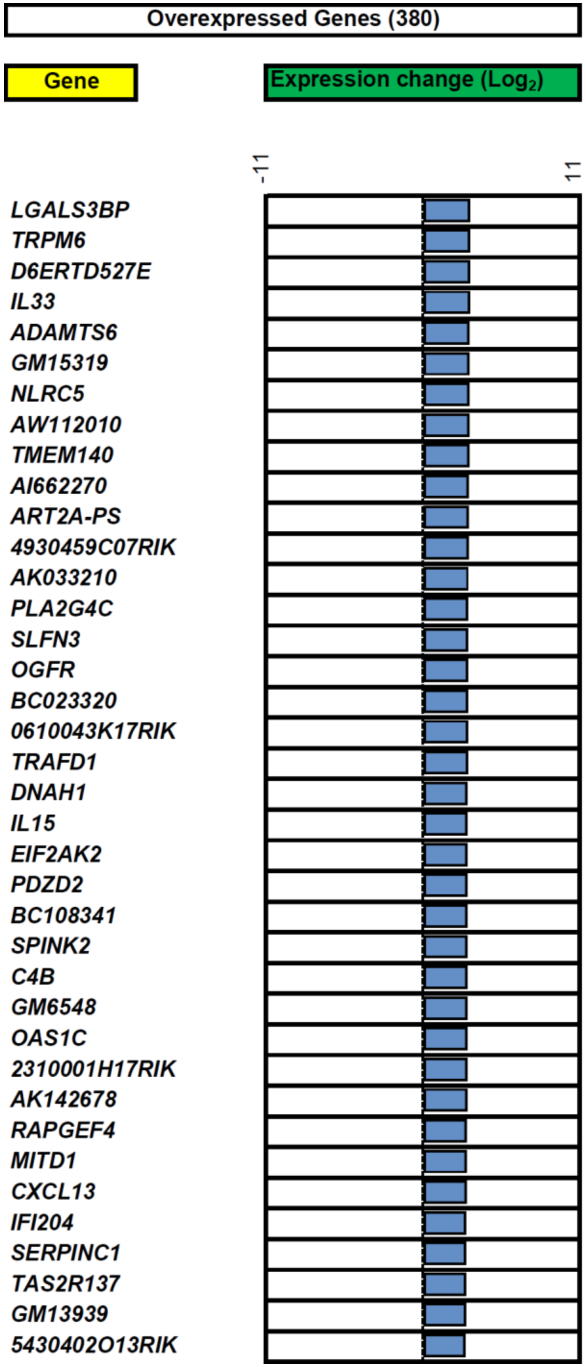

Figure C (cont’d)

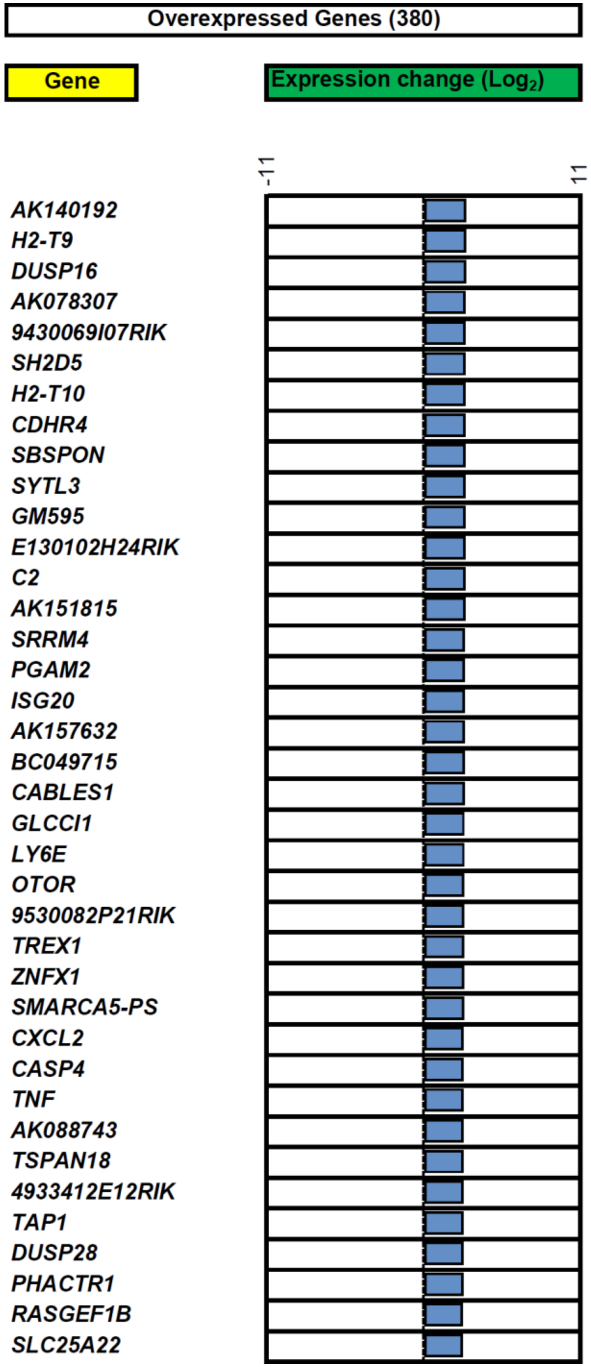

Figure C (cont’d)

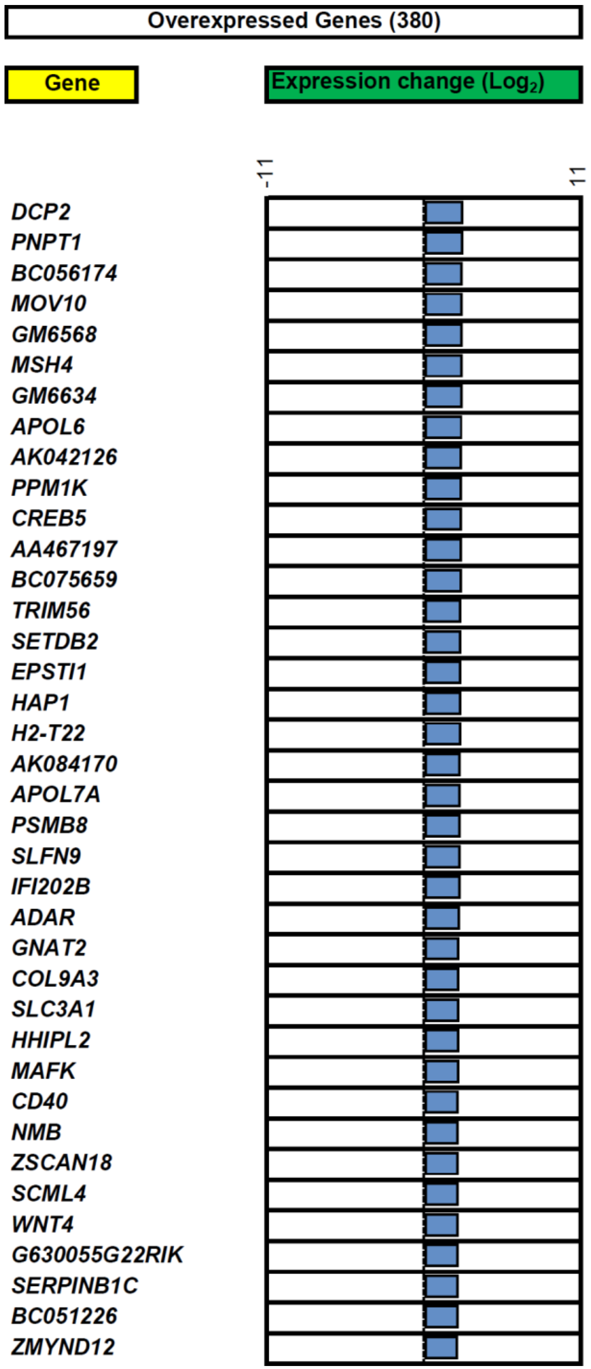

Figure C (cont'd)

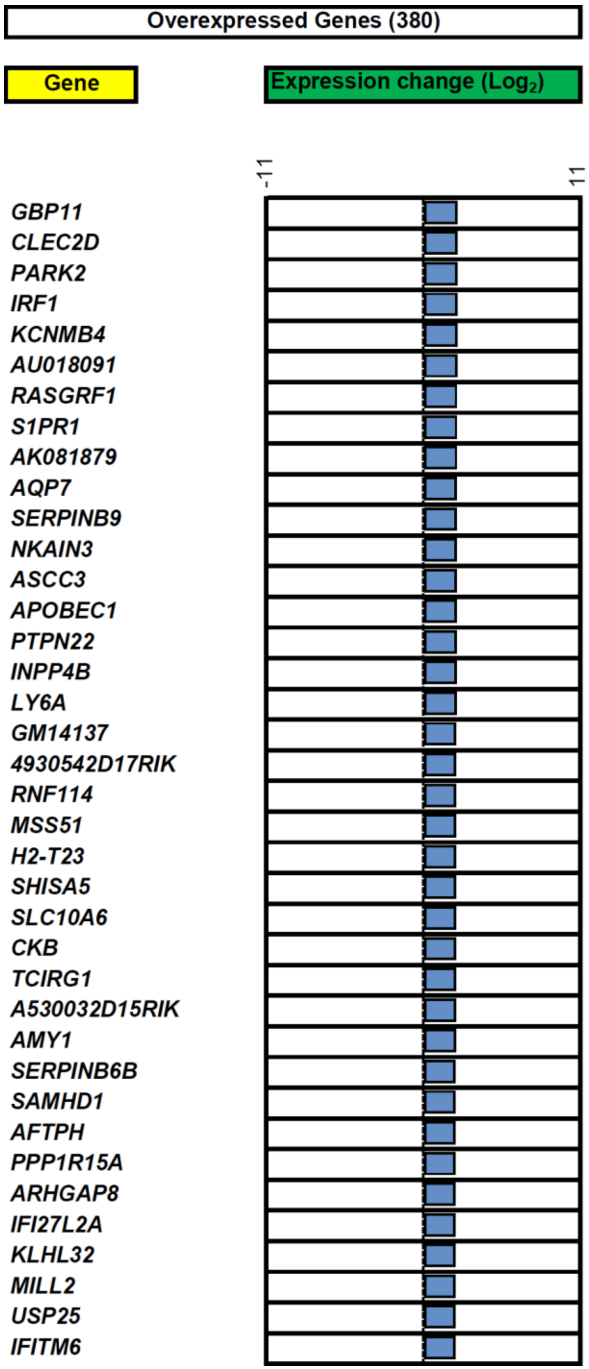

Figure C (cont'd)

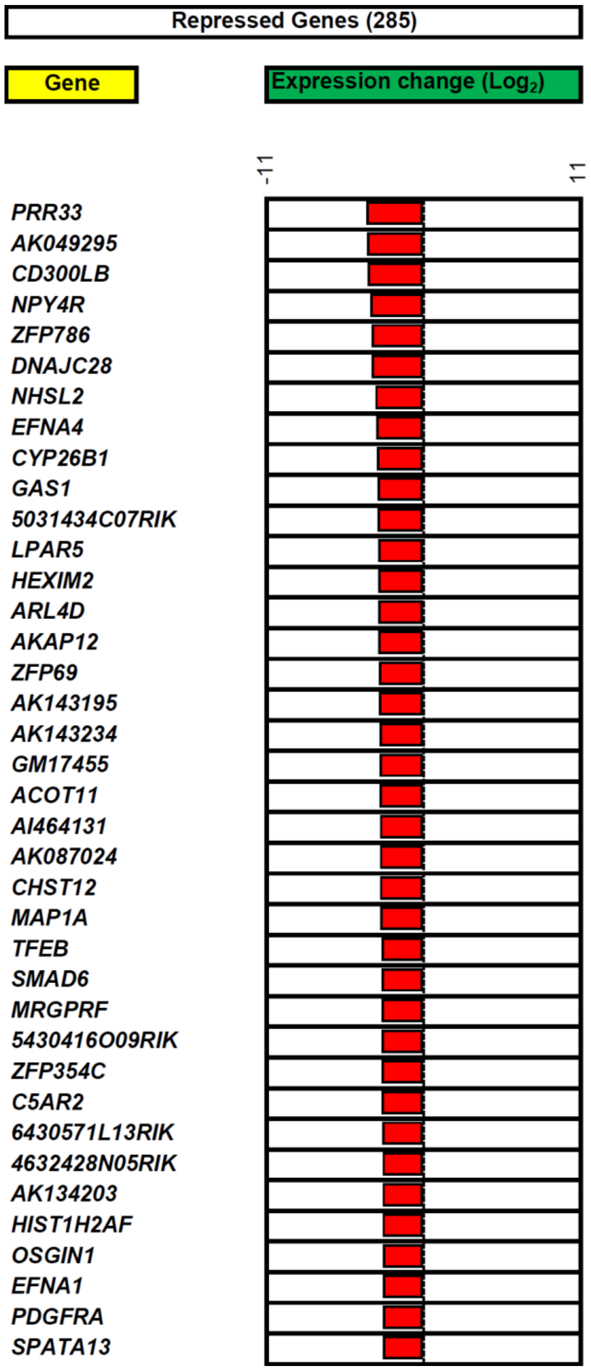

Figure C (cont'd)

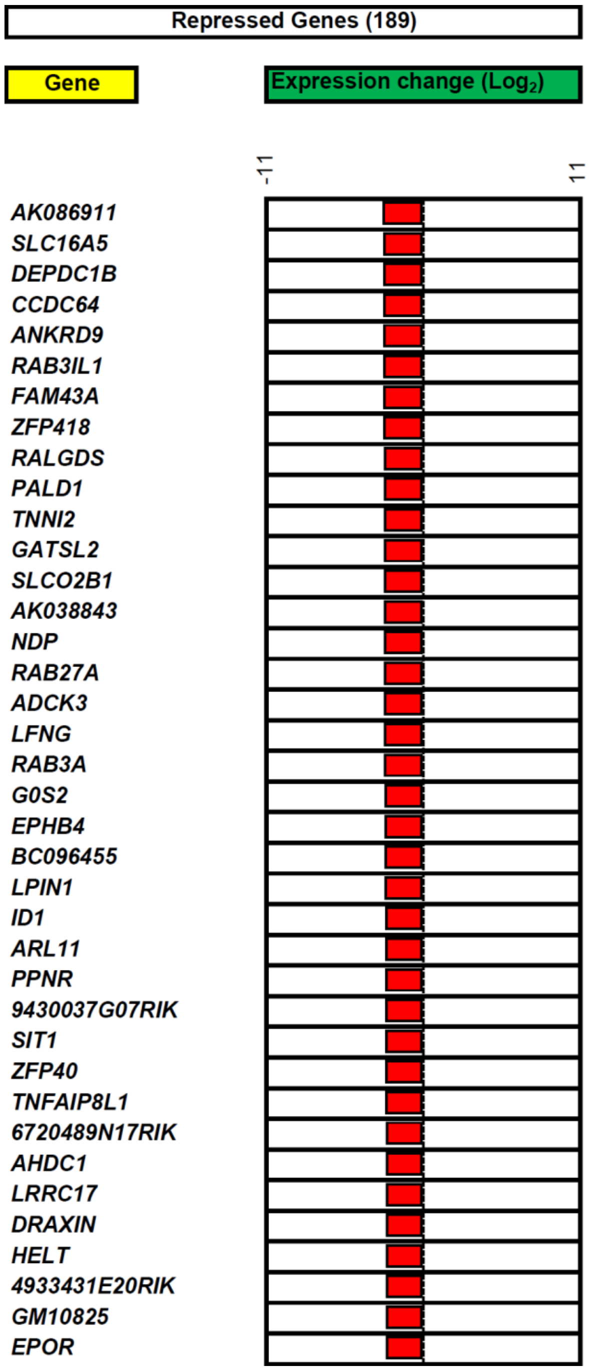

Figure C (cont’d)

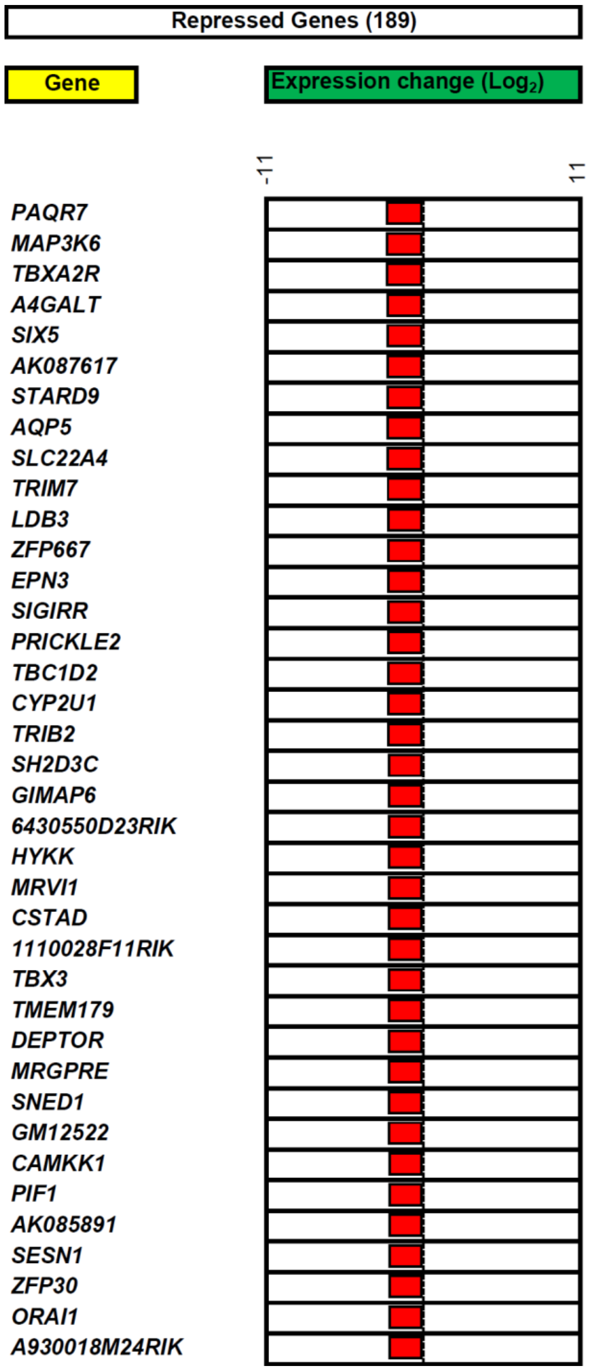

Figure C (cont'd)

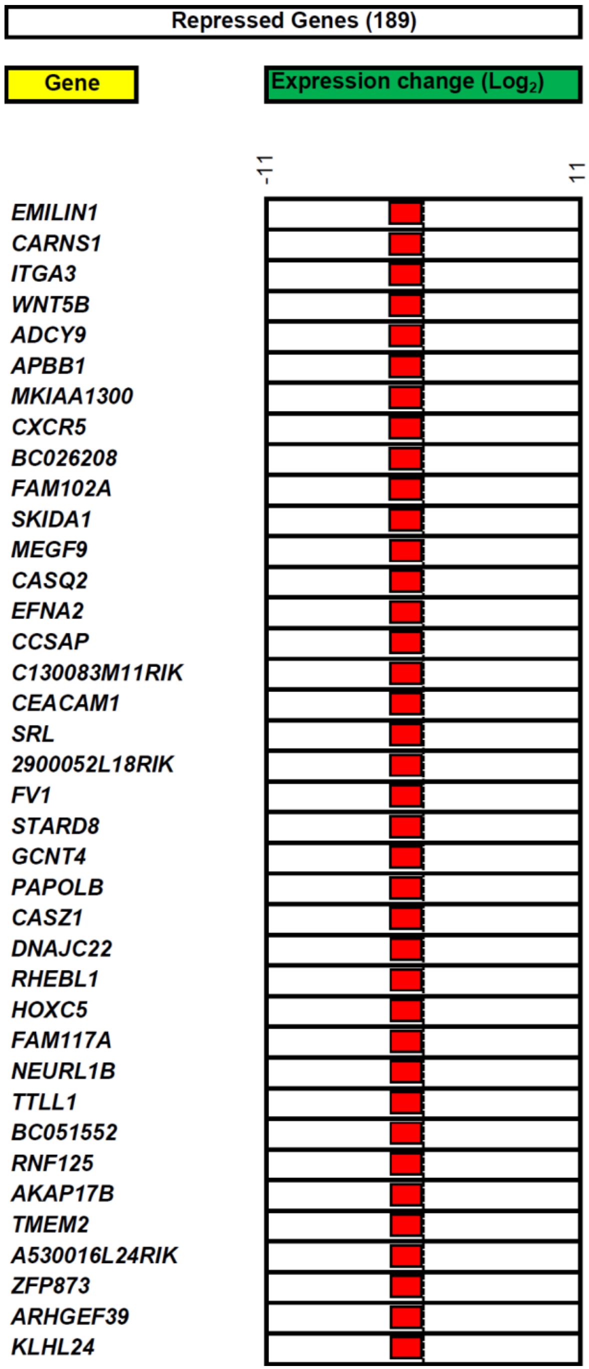

Figure C (cont'd)

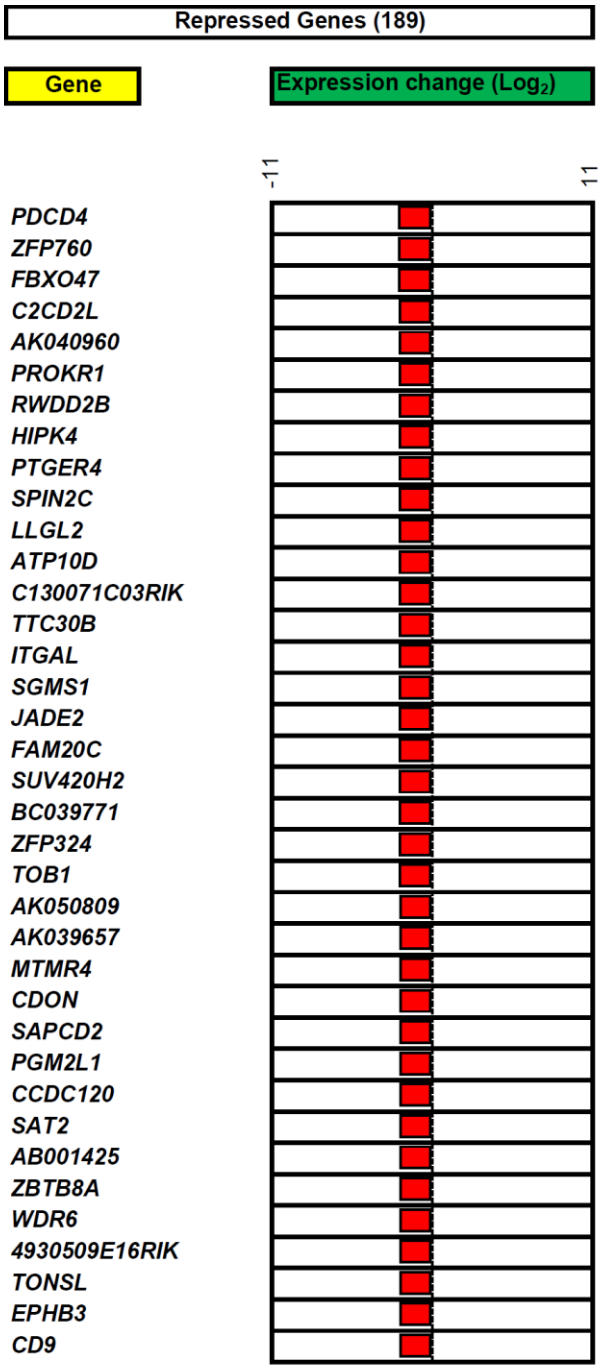

Figure C (cont’d)

Figure D. Overexpressed genes interaction network during reovirus infection.

Interaction network for the 380 cellular genes for which the expression was upregulated upon viral infection. The network was determined by uploading the genes into STRING. Solid lines: direct known interactions; dashed lines: suspected or indirect interactions.

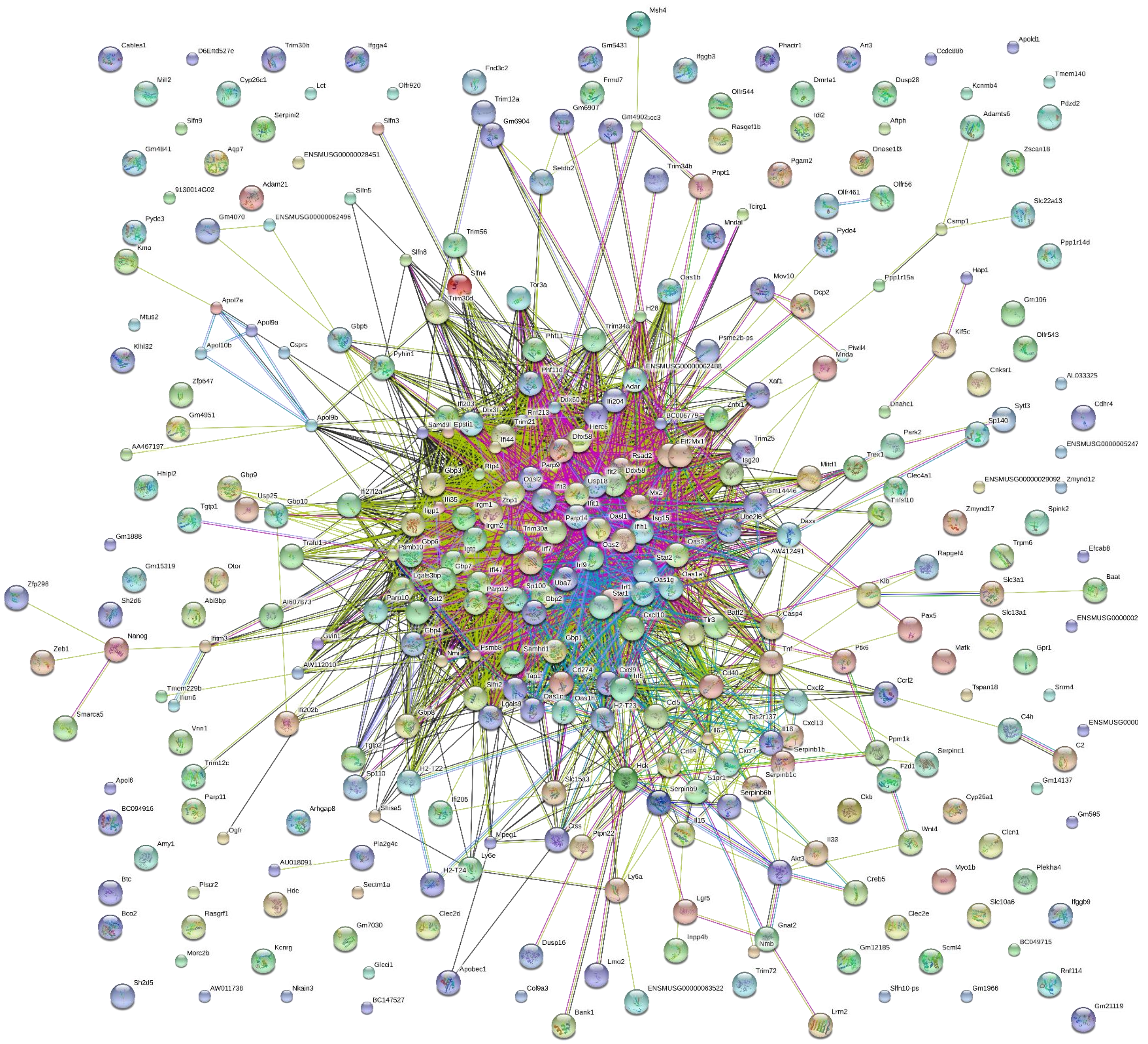

Msh4
Cables1
D6Ertd527e
Trim30b
Ifgga4
Gm5431
Phactr1
Art3
Ccdc88b
Apold1
Mill2
Cyp26c1
Lct
Olfr920
Fnd3c2
Ifggb3
Frmd7
Olfr544
Dmrta1
Dusp28
Kcnmb4
Tmem140
Slfn9
Serpini2
Slfn3
Trim12a
Gm6904
Gm6907
Rasgef1b
Idi2
Aftph
Adamts6
Pdzd2
Gm4841
Aqp7
ENSMUSG00000028451
Setdb2
Trim34b
Pnpt1
Pgam2
Dnase1l3
Zscan18
9130014G02
Adam21
Slfn5
Olfr461
Olfr56
Slc22a13
Pydc3
Gm4070
ENSMUSG00000062496
Tcirg1
Mndal
Csmp1
Kmo
Trim56
Slfn8
Pydc4
Ppp1r14d
Slfn4
Trim30d
Tor3a
Oas1b
Mov10
Dcp2
Ppp1r15a
Hap1
Apol7a
H28
Gm106
Mtus2
Apol9a
Gbp5
Trim34a
Psme2b-ps
Kif5c
Olfr543
Klhl32
Apol10b
Csprs
Pyhin1
Phf11
Piwil4
Mnda
Zfp647
Phf11d
ENSMUSG00000062488
Xaf1
Cnksr1
AL033325
Ifi203
Ddx60
Adar
Ifi204
Znfx1
Dnahc1
Sytl3
Sp140
Cdhr4
Gm4951
Apol9b
Samd9l
Epsti1
Dtx3l
Rnf213
Trim21
Herc6
BC006779
Park2
AA467197
Ifi44
Dhx58
Eif2ak2
Mx1
Trim25
Rsad2
Isg20
Trex1
Mitd1
Clec4a1
ENSMUSG00000029092
Zmynd12
Hhipl2
Gbp9
Parp9
Ddx58
Ifit2
Oasl2
Gbp3
Rtp4
Usp18
Ifit3
Usp25
Gbp10
Ifi27l2a
Il15
Zbp1
Mx2
Gm14446
Tnfsf10
Zmynd17
Spink2
Tgtp1
Iigp1
Irgm1
Ifit1
Parp14
Oasl1
Isg15
Ube2l6
Daxx
Gm1888
Irgm2
Trim30a
Ifih1
Oas3
AW412491
Trpm6
Traf1
Psmb10
Gbp6
Igtp
Oas2
Irf7
Stat2
Rapgef4
Efcab8
Gm15319
Gbp7
Ifi47
Irf9
Oas1a
Klb
Slc3a1
Baat
Zfp296
Lgals3bp
Uba7
Oas1g
Sh2d6
Abi3bp
Parp10
Bst2
Parp12
Sp100
Irf1
Stat1
AI607873
Gbp2
Cxcl10
Tlr3
Batf2
Casp4
Pax5
Slc13a1
Nanog
Ifitm3
Gvin1
Gbp4
Gbp1
Tnf
Ptk6
Mafk
Gpr1
Zeb1
Nmi
Psmb8
Samhd1
AW112010
Tap1
Cd274
Cxcl9
Il15
Ccl4
Ccrl2
Tmem229b
Ifitm6
Slfn2
Lgals9
Oas1c
Oas1h
H2-T23
Ccl5
Cxcl2
Tspan18
Srrm4
Smarca5
Ifi202b
Gbp8
Tas2r137
Il18
Cxcl13
C4b
ENSMUSG0000
Vnn1
Il6
Cxcr7
Serpinb1b
Ppm1k
C2
Trim12c
Tgtp2
Cd69
Serpinb1c
Serpinc1
Fzd1
Sp110
H2-T22
Slc15a3
Hck
S1pr1
Serpinb9
Serpinb6b
Gm14137
Apol6
Ifi205
Ckb
Cyp26a1
BC094916
Parp11
Arhgap8
Shisa5
Il15
Gm595
Oglr
Mpeg1
Ctss
Ptpn22
Wnt4
Clcn1
Amy1
Pla2g4c
Ly6e
Il33
AU018091
Akt3
Btc
H2-T24
Plscr2
Secim1a
Ly6a
Creb5
Myo1b
Plekha4
Hdc
Lgr5
Slc10a6
Ifggb9
Bco2
Rasgrf1
Clec2d
Clec2e
Gm7030
Gnat2
Inpp4b
Dusp16
Nmb
Gm12185
BC049715
Morc2b
Kcnrg
Scml4
Lmo2
Glcci1
Apobec1
Trim72
Rnf114
Sh2d5
AW011738
Nkain3
Col9a3
ENSMUSG00000063522
Slfn10-ps
Gm1966
BC147527
Bank1
Lrrn2
Gm21119

Figure D

Figure E. 240 ASEs are modified during viral infection with reovirus.

Heatmap representation with a sequential color scale of the 240 ASEs that are differentially spliced upon viral infection. RNA sequencing was done in triplicate for both the uninfected (mock) and infected cells. Darker shades indicates high ΔPSI values and lighter shades indicates low ΔPSI values between infected and mock cells. More than one ASEs can impact the same gene.

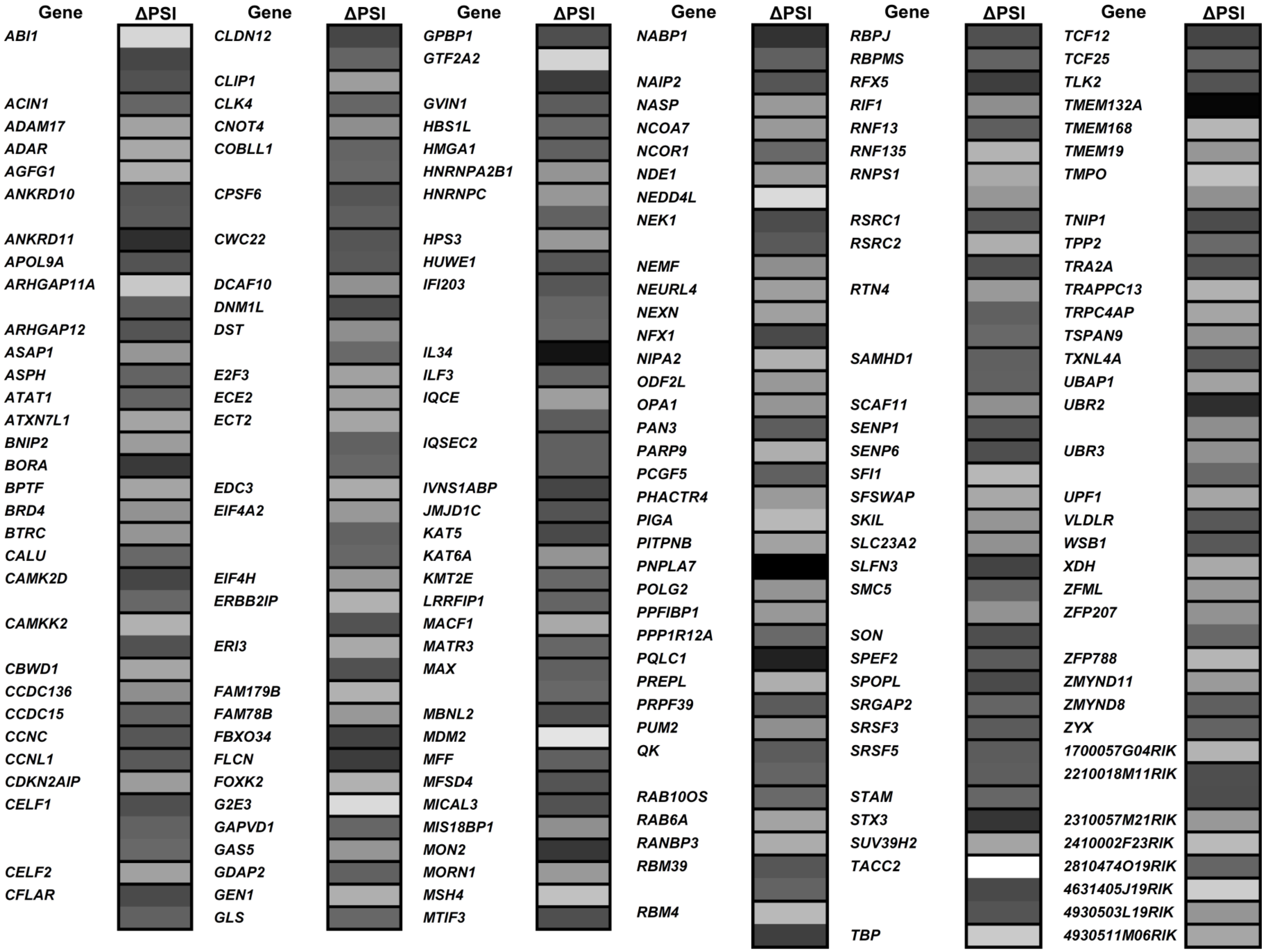
Gene
ΔPSI
ABI1
ACIN1
ADAM17
ADAR
AGFG1
ANKRD10
ANKRD11
APOL9A
ARHGAP11A
ARHGAP12
ASAP1
ASPH
ATAT1
ATXN7L1
BNIP2
BORA
BPTF
BRD4
BTRC
CALU
CAMK2D
CAMKK2
CBWD1
CCDC136
CCDC15
CCNC
CCNL1
CDKN2AIP
CELF1
CELF2
CFLAR
Gene
ΔPSI
CLDN12
CLIP1
CLK4
CNOT4
COBLL1
CPSF6
CWC22
DCAF10
DNM1L
DST
E2F3
ECE2
ECT2
EDC3
EIF4A2
EIF4H
ERBB2IP
ERI3
FAM179B
FAM78B
FBXO34
FLCN
FOXK2
G2E3
GAPVD1
GAS5
GDAP2
GEN1
GLS
Gene
ΔPSI
GPBP1
GTF2A2
GVIN1
HBS1L
HMGA1
HNRNPA2B1
HNRNPC
HPS3
HUWE1
IFI203
IL34
ILF3
IQCE
IQSEC2
IVNS1ABP
JMJD1C
KAT5
KAT6A
KMT2E
LRRFIP1
MACF1
MATR3
MAX
MBNL2
MDM2
MFF
MFSD4
MICAL3
MIS18BP1
MON2
MORN1
MSH4
MTIF3
Gene
ΔPSI
NABP1
NAIP2
NASP
NCOA7
NCOR1
NDE1
NEDD4L
NEK1
NEMF
NEURL4
NEXN
NFX1
NIPA2
ODF2L
OPA1
PAN3
PARP9
PCGF5
PHACTR4
PIGA
PITPNB
PNPLA7
POLG2
PPFIBP1
PPP1R12A
PQLC1
PREPL
PRPF39
PUM2
QK
RAB10OS
RAB6A
RANBP3
RBM39
RBM4
Gene
ΔPSI
RBPJ
RBPMS
RFX5
RIF1
RNF13
RNF135
RNPS1
RSRC1
RSRC2
RTN4
SAMHD1
SCAF11
SENP1
SENP6
SFI1
SFSWAP
SKIL
SLC23A2
SLFN3
SMC5
SON
SPEF2
SPOPL
SRGAP2
SRSF3
SRSF5
STAM
STX3
SUV39H2
TACC2
TBP
Gene
ΔPSI
TCF12
TCF25
TLK2
TMEM132A
TMEM168
TMEM19
TMPO
TNIP1
TPP2
TRA2A
TRAPPC13
TRPC4AP
TSPAN9
TXNL4A
UBAP1
UBR2
UBR3
UPF1
VLDLR
WSB1
XDH
ZFML
ZFP207
ZFP788
ZMYND11
ZMYND8
ZYX
1700057G04RIK
2210018M11RIK
2310057M21RIK
2410002F23RIK
2810474O19RIK
4631405J19RIK
4930503L19RIK
4930511M06RIK

Figure E

Figure F. Profiling of the cellular alternative splicing landscape during reovirus infection.

(A) Interaction network for genes encoding the 240 differentially spliced transcripts upon viral infection. The network was determined by uploading the corresponding genes into STRING. Solid lines: direct known interactions; dashed lines: suspected or indirect interactions.

(B) Gene ontology analysis of the 240 differentially spliced transcripts upon viral infection. The corresponding genes were imported into the DAVID gene ontology suite of programs at the NIAID. Ontological functions were determined determined for biological processes using all ASEs caracterized as background.

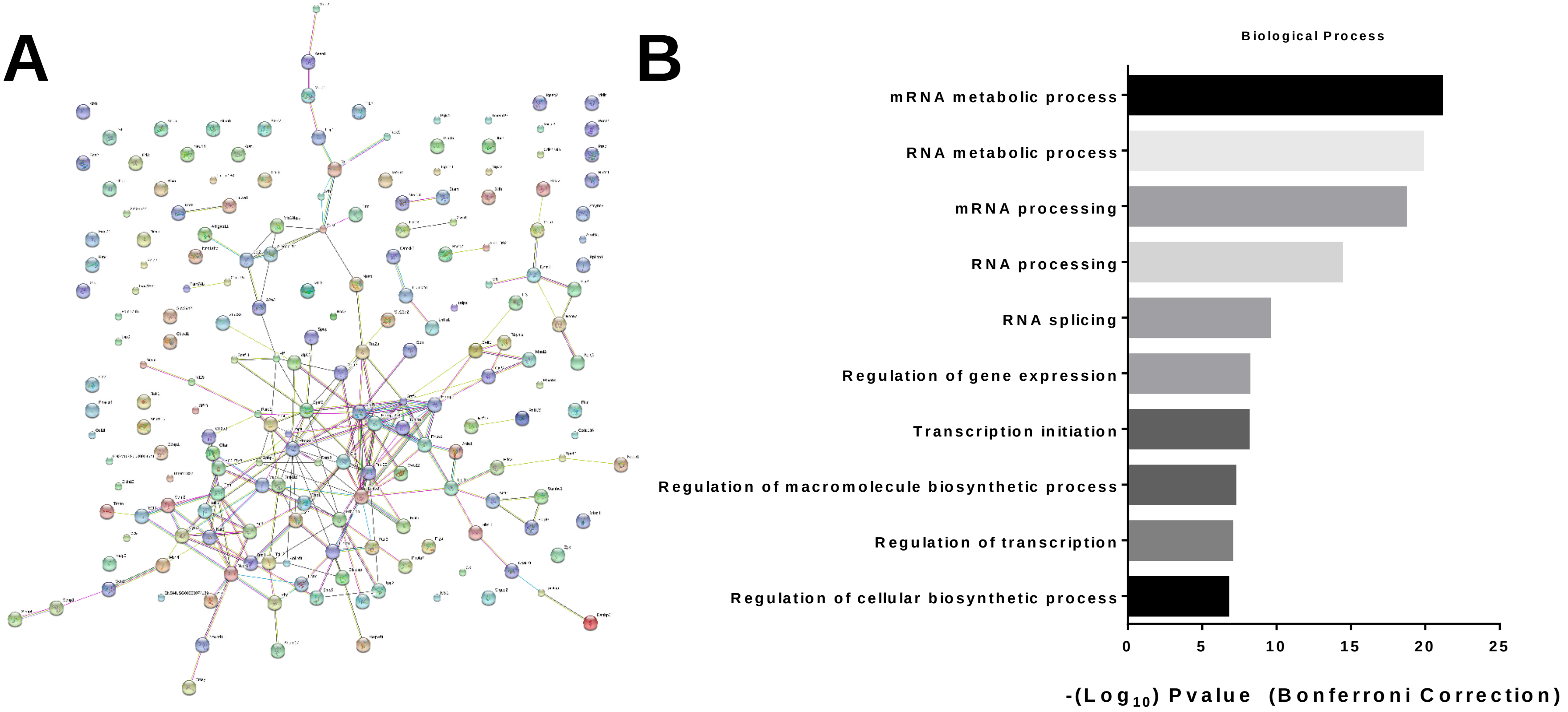
A
B
Biological Process
mRNA metabolic process
RNA metabolic process
mRNA processing
RNA processing
RNA splicing
Regulation of gene expression
Transcription initiation
Regulation of macromolecule biosynthetic process
Regulation of transcription
Regulation of cellular biosynthetic process
0
5
10
15
20
25
-(Log10) Pvalue (Bonferroni Correction)

Figure F

Figure G. Additional validation of ASEs dysregulated in infected cells.

(A, C, E) Overview of two isoforms encoded by the *HNRNPA2B1, EIF4A2,* and *IL34* genes. Exons are depicted in red and and the intervening introns are shown as thin black lines (not to scale). The primers used to detect the ASEs by RT-PCR assays are shown in gray and the sizes of the expected amplicons (162 nt and 282 nt, 112 nt and 219 nt, 256 nt and 374 nt, respectively) are also indicated. The genomic coordinates of the two isoforms are also indicated.

(B, D, F) Cellular mRNAs isolated from both uninfected and infected cells were analyzed by RT-PCR using specific primers to detect both forms of the ASE encoded on various genes. The amplified products were analyzed by automated chip-based microcapillary electrophoresis. Capillary electrophoregrams of the PCR reactions are shown. The positions and the amplitude of the detected amplicons are highlighted by red boxes. The positions of the internal markers are also indicated.

A

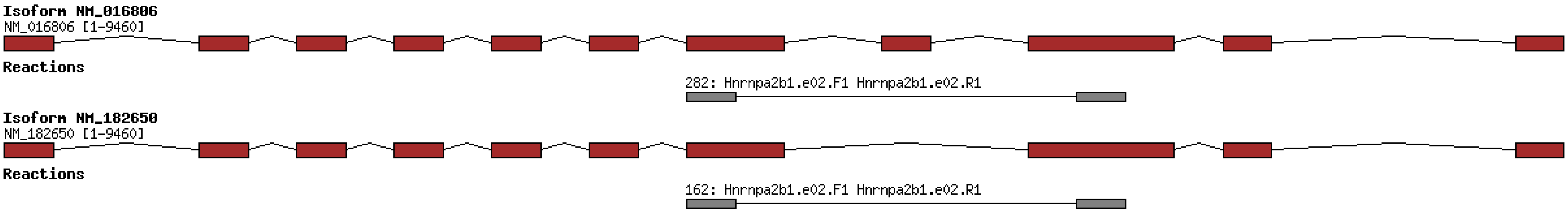

B

HNRNPA2B1 - Uninfected

HNRNPA2B1 - Infected

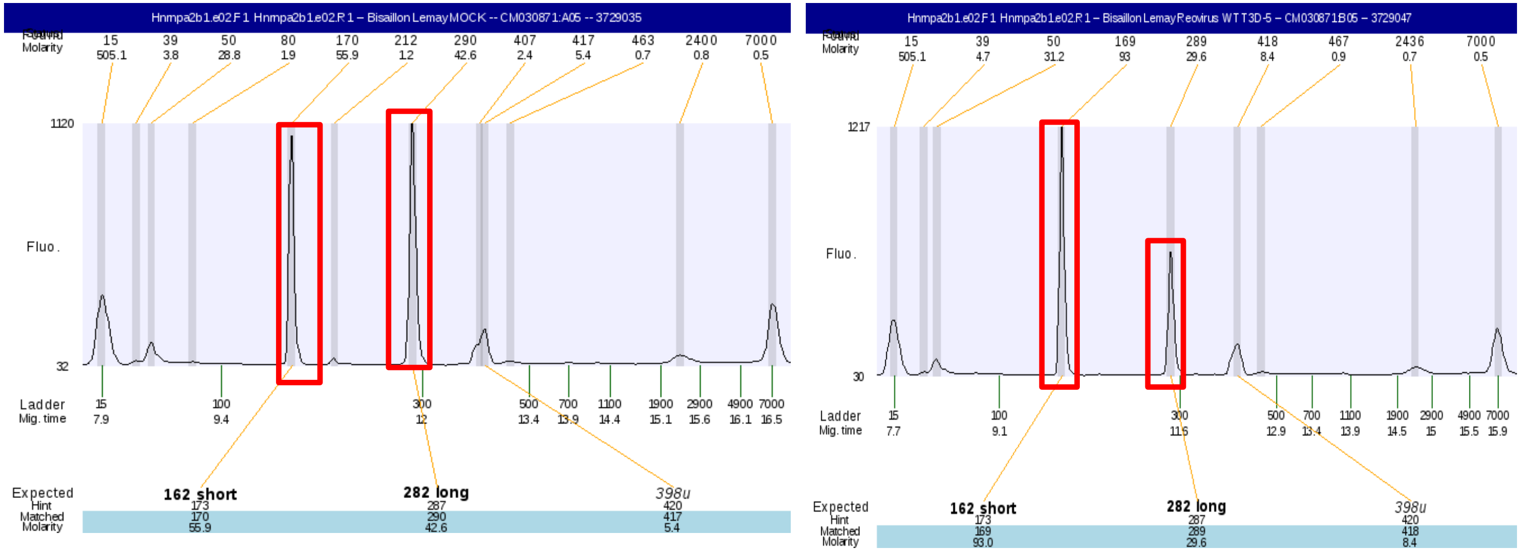

Figure G

C

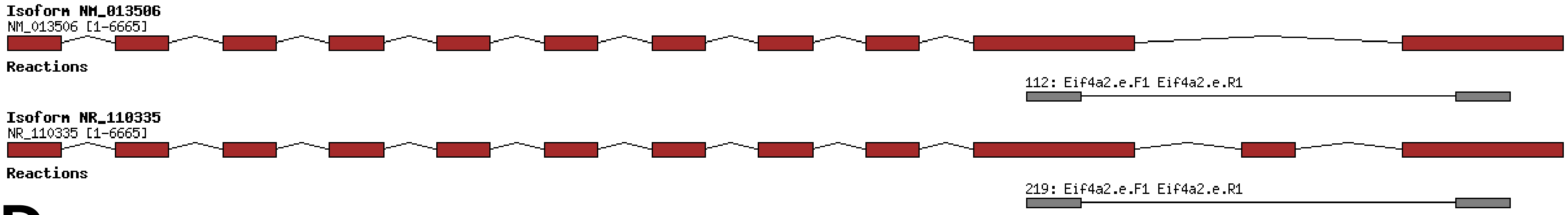

D

EIF4A2 - Uninfected

EIF4A2 – Infected

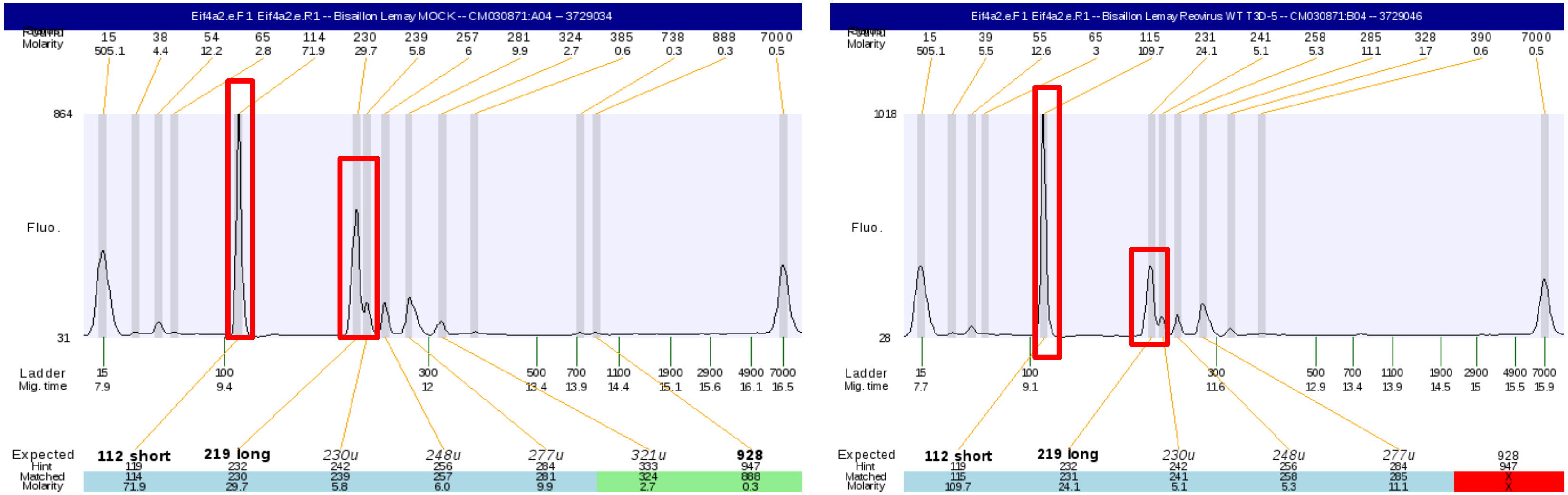

E

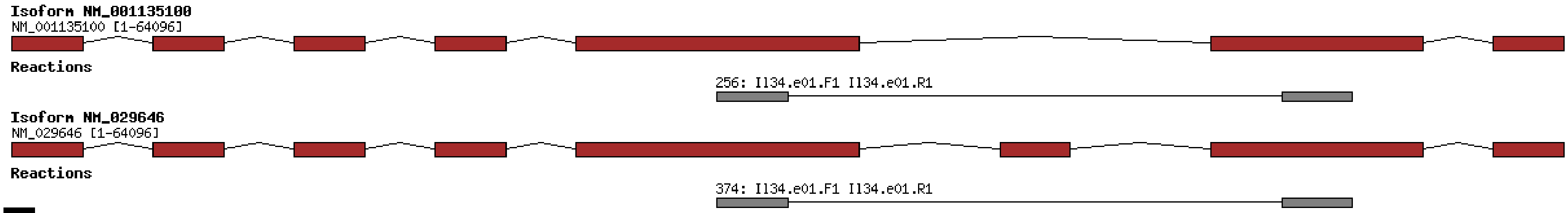

F

IL34 – Uninfected

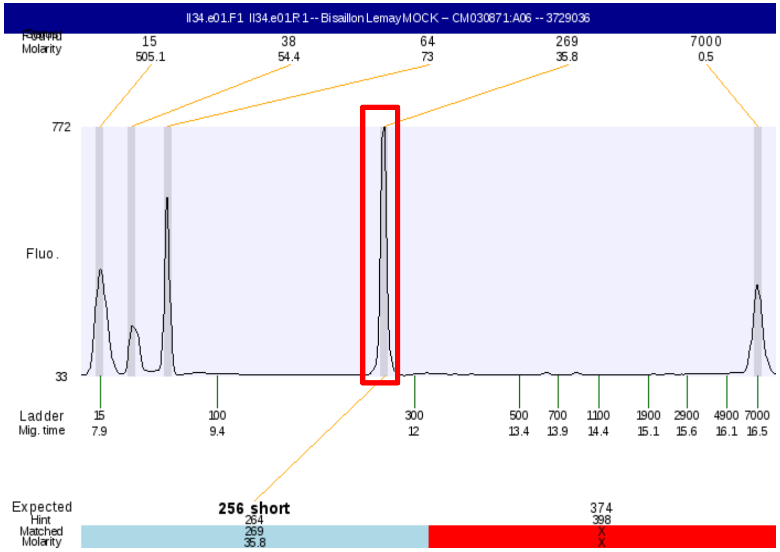

IL34 – Infected

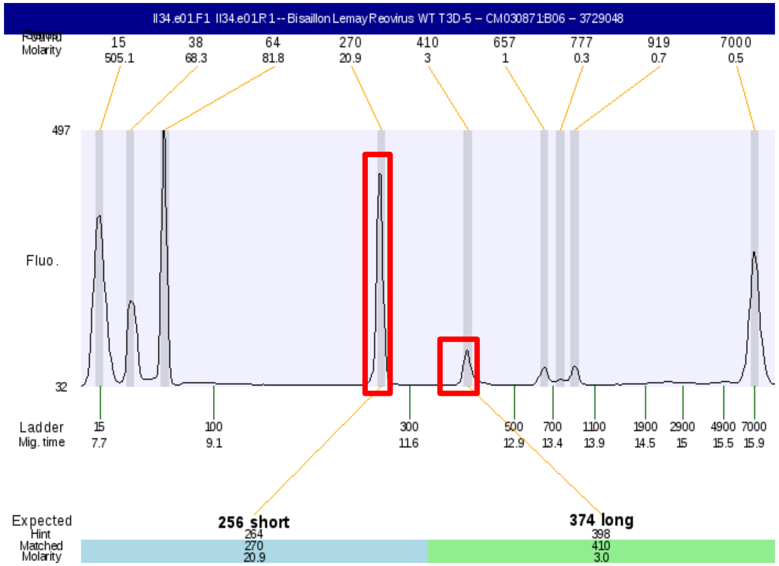

Figure G (cont'd)

Figure H. List of the differentially ASEs associated with viral infection.

The list displays the 240 differentially spliced transcripts with the corresponding Delta PSI values, the associated gene expression (in Log_2), and the associated biological processes.

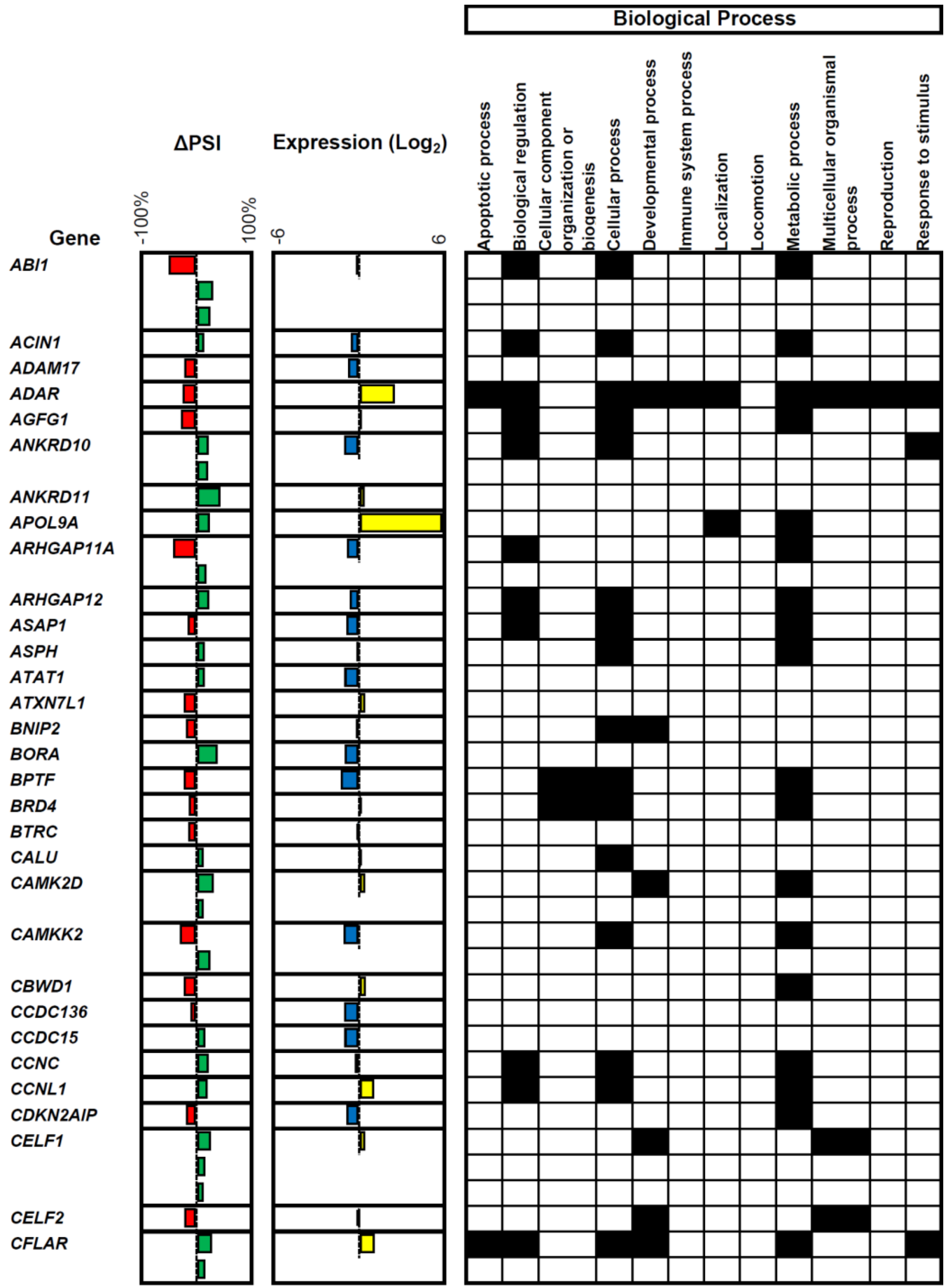

Biological Process
ΔPSI
Expression (Log2)
Gene
-100%
100%
-6
6
Apoptotic process
Biological regulation
Cellular component organization or biogenesis
Cellular process
Developmental process
Immune system process
Localization
Locomotion
Metabolic process
Multicellular organismal process
Reproduction
Response to stimulus
ABI1
ACIN1
ADAM17
ADAR
AGFG1
ANKRD10
ANKRD11
APOL9A
ARHGAP11A
ARHGAP12
ASAP1
ASPH
ATAT1
ATXN7L1
BNIP2
BORA
BPTF
BRD4
BTRC
CALU
CAMK2D
CAMKK2
CBWD1
CCDC136
CCDC15
CCNC
CCNL1
CDKN2AIP
CELF1
CELF2
CFLAR

Figure H

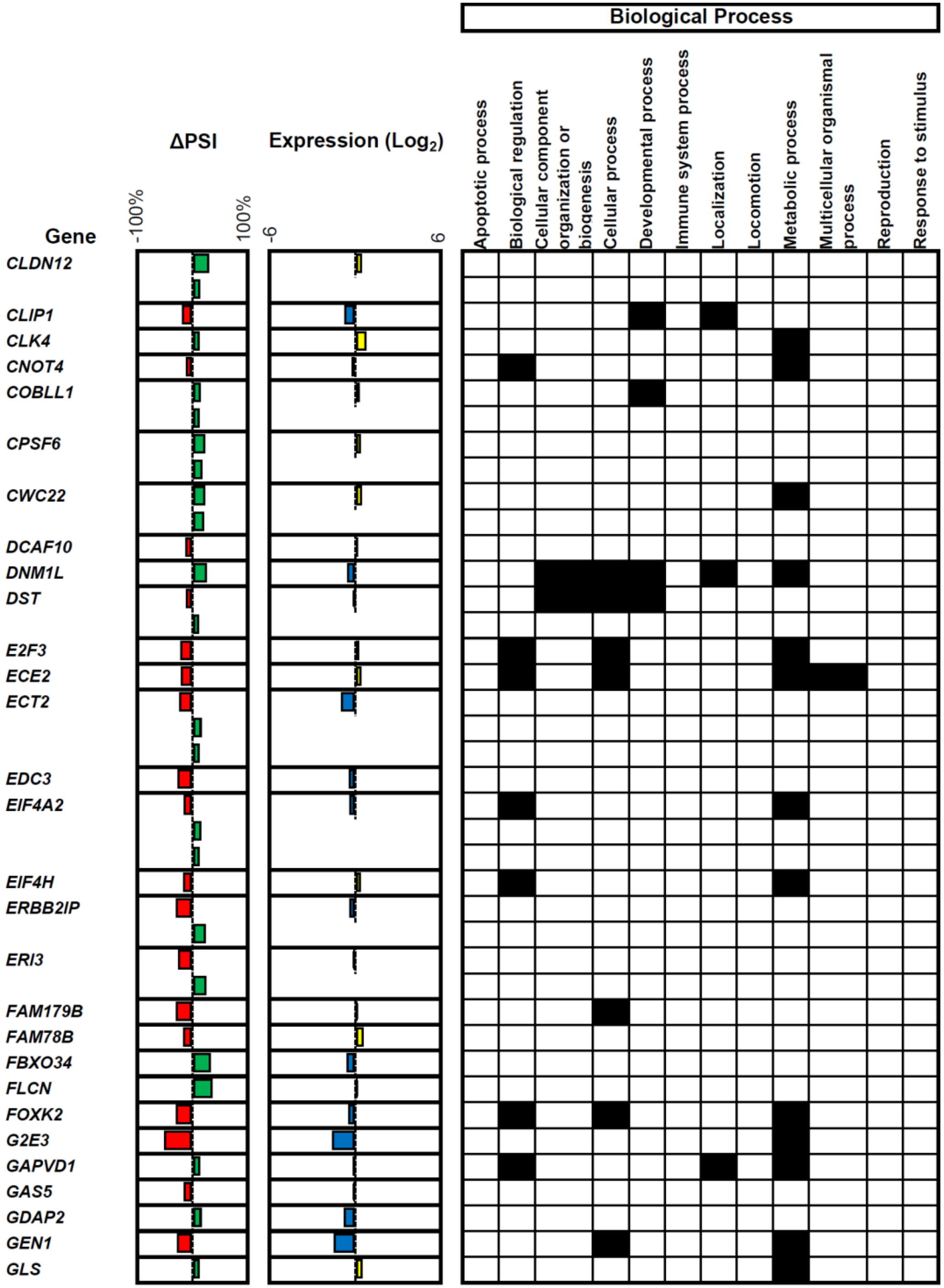

Figure H (cont’d)

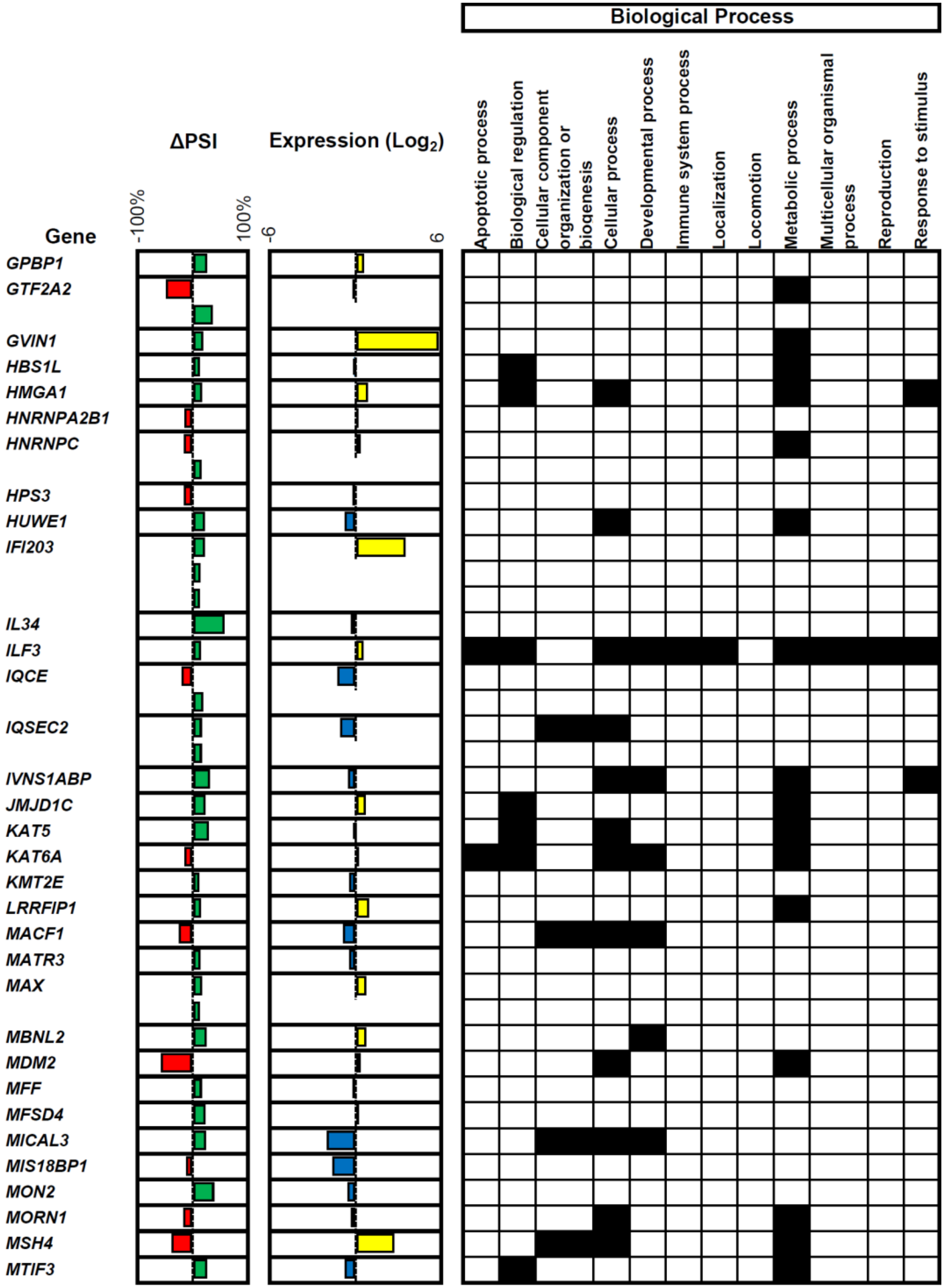

Figure H (cont’d)

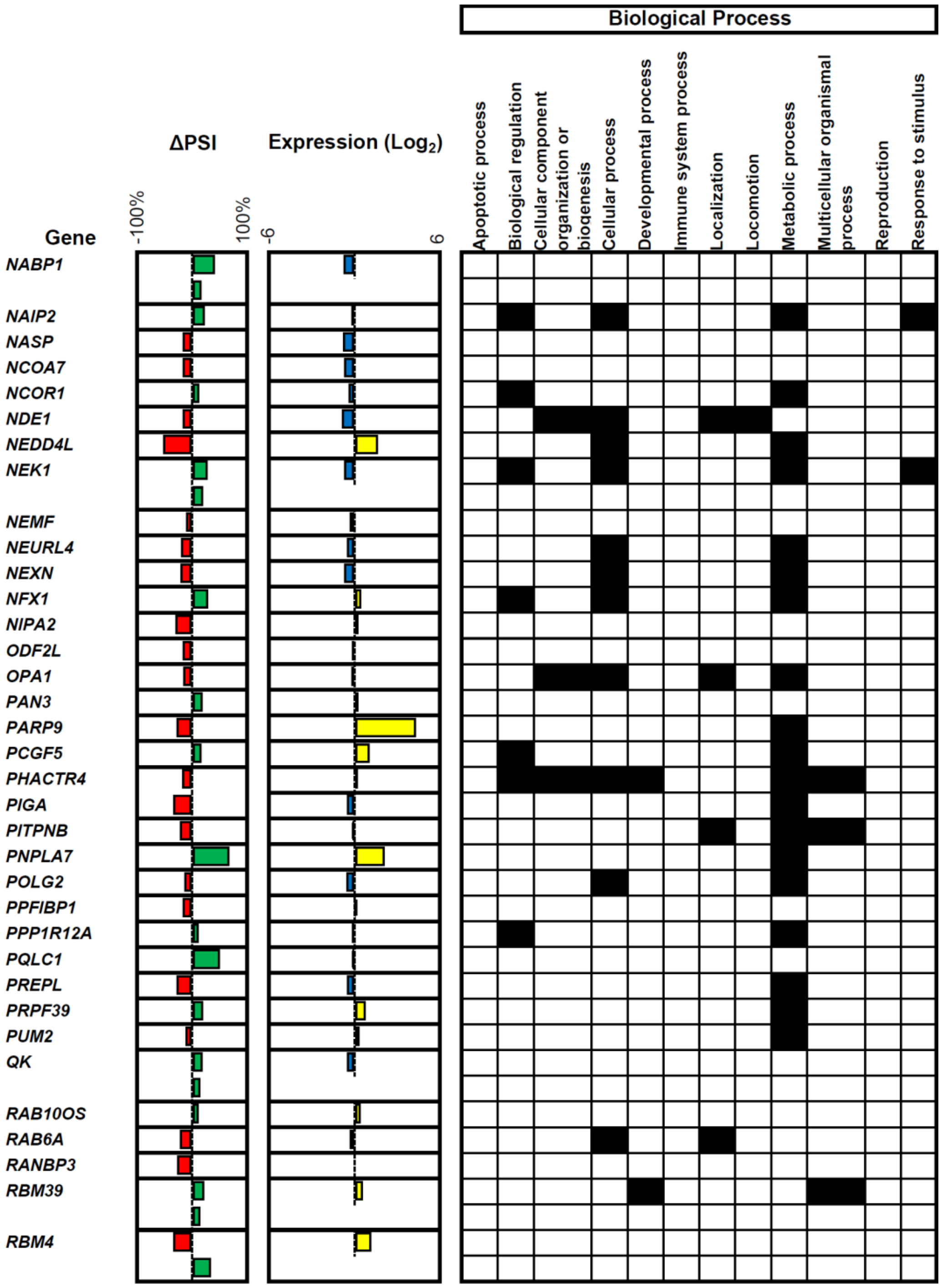

Figure H (cont'd)

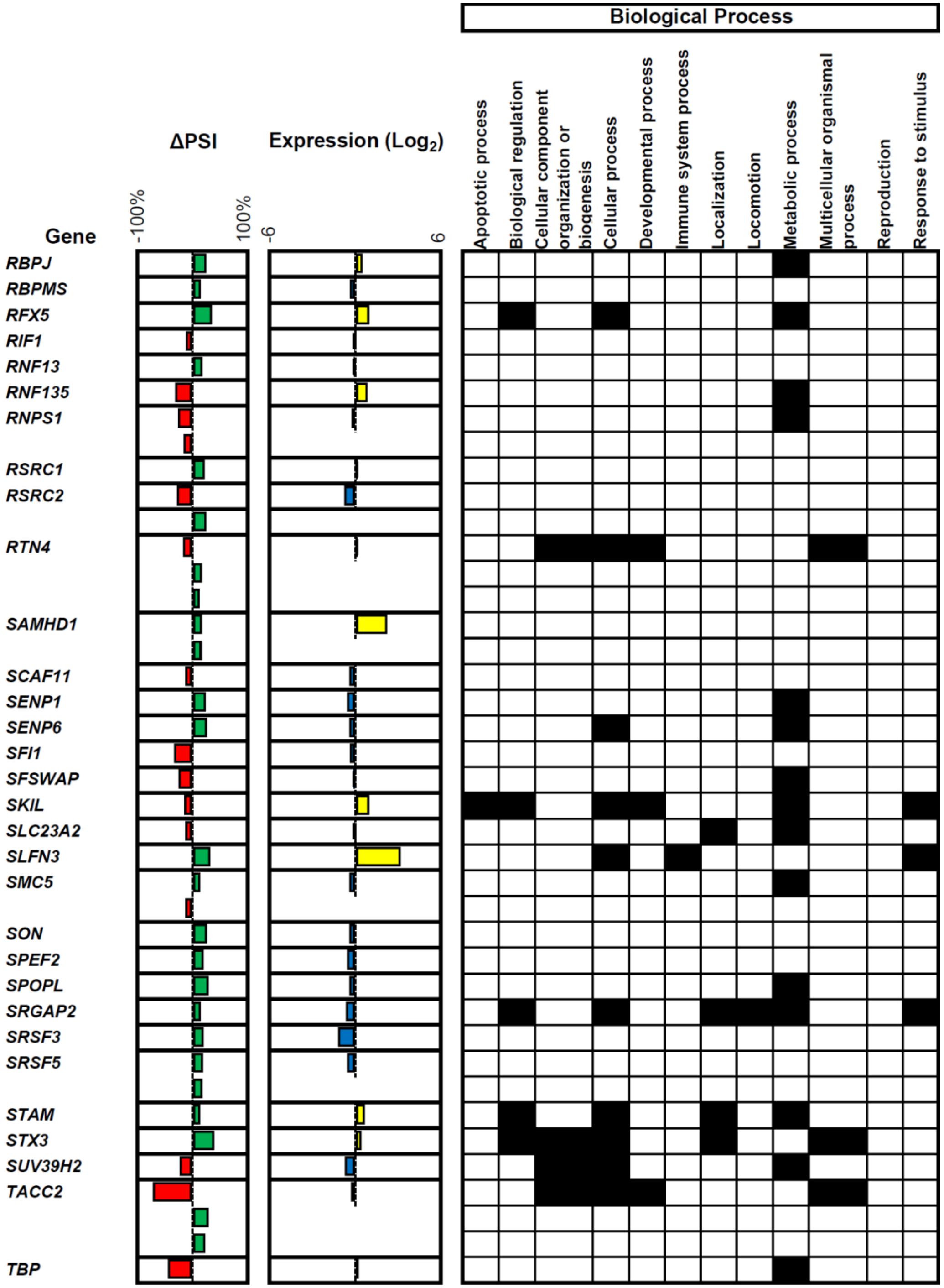

Figure H (cont'd)

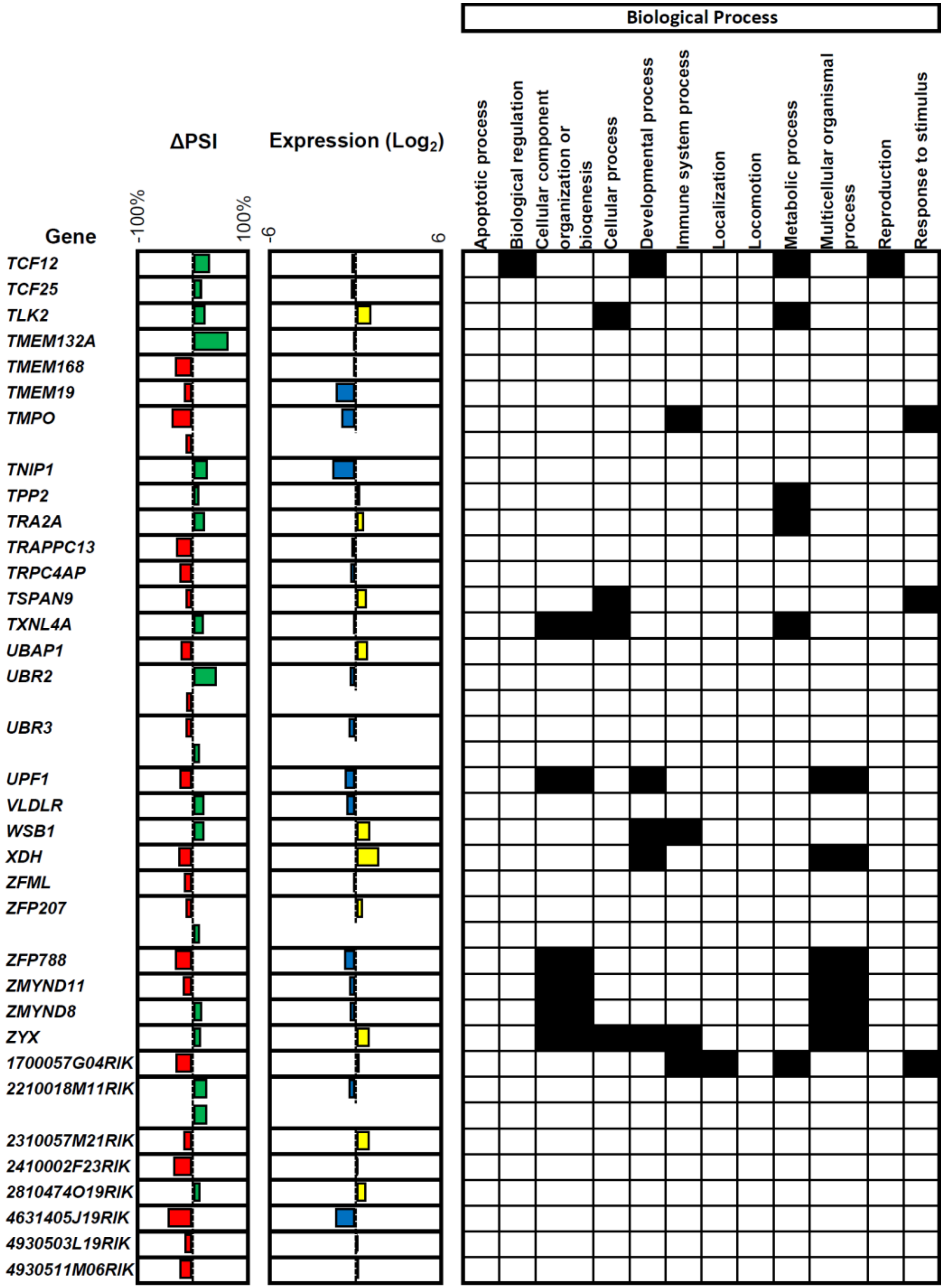

Figure H (cont'd)

Figure I. Common ASEs modified between WT reovirus and P4L-12 mutant show a strong correlation.

Correlation between PSI values for WT and P4L-12 mutant reovirus.

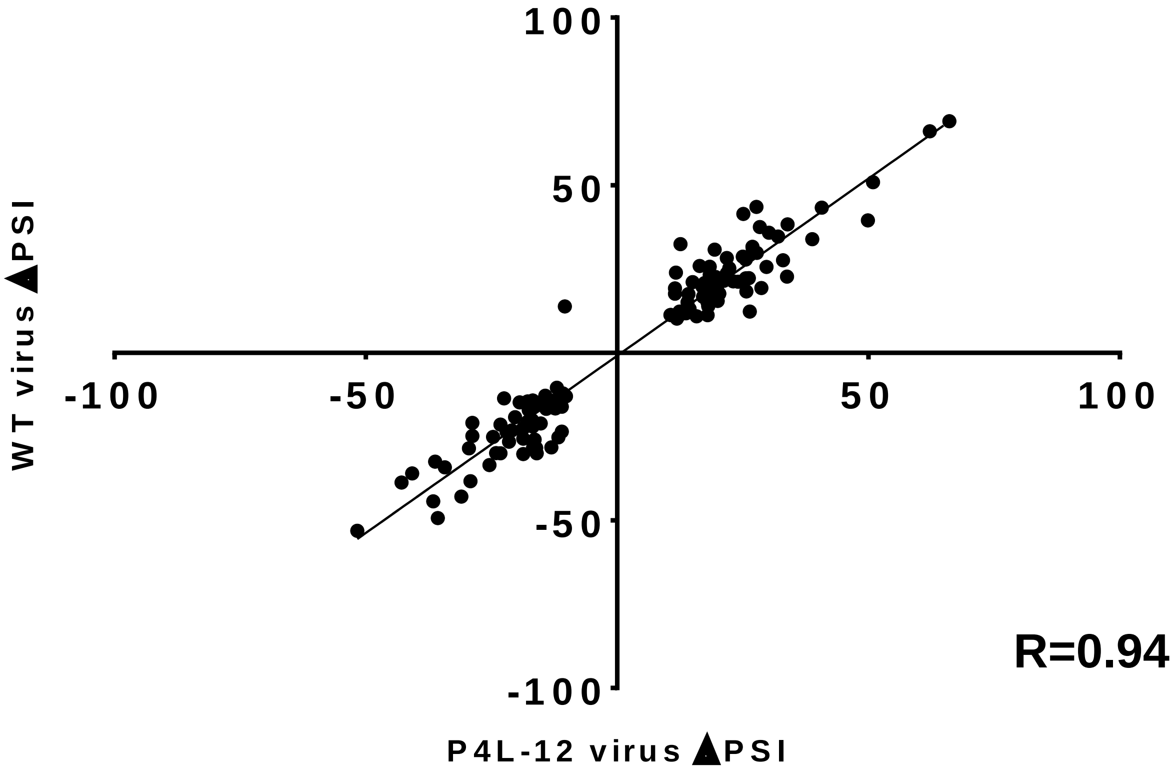

Figure I

Figure J. Analysis of the 240 differnentially spliced transcripts highlights a consensus sequence.

A significant enrichment of a 41 nt motif in the vicinity of many splice regions was found in transcripts for which AS was altered upon viral infection. The motif was found in 93 of the 240 transcripts that were differentially spliced upon viral infection (e value = $1{,}3e\text{-}10^{49}$). The diffentially spliced exon and its flanking introns were used to search for this motif.

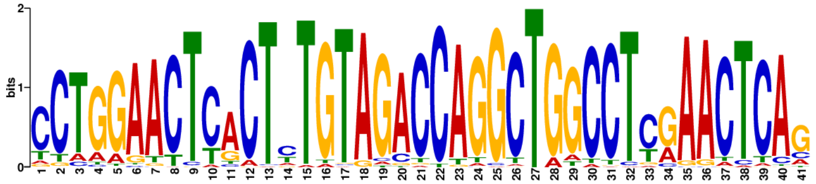

bits
2
1
0
1 2 3 4 5 6 7 8 9 10 11 12 13 14 15 16 17 18 19 20 21 22 23 24 25 26 27 28 29 30 31 32 33 34 35 36 37 38 39 40 41

Figure J
